# Prediction of preeclampsia risk in first time pregnant women: Metabolite biomarkers for a clinical test

**DOI:** 10.1371/journal.pone.0244369

**Published:** 2020-12-28

**Authors:** Louise C. Kenny, Grégoire Thomas, Lucilla Poston, Jenny E. Myers, Nigel A. B. Simpson, Fergus P. McCarthy, Leslie W. Brown, Alison E. Bond, Robin Tuytten, Philip N. Baker, on behalf of the Screening for Pregnancy Endpoints Consortium

**Affiliations:** 1 Faculty of Health & Life Sciences, University of Liverpool, Liverpool, United Kingdom; 2 SQU4RE, Lokeren, Belgium; 3 Metabolomic Diagnostics, Cork, Ireland; 4 Department of Women and Children’s Health, King’s College London, London, United Kingdom; 5 Maternal & Fetal Health Research Centre, Manchester Academic Health Science Centre, University of Manchester, Manchester, United Kingdom; 6 Department of Women’s and Children’s Health, University of Leeds, Leeds, United Kingdom; 7 Irish Centre for Fetal and Neonatal Translational Research, University College Cork, Cork, Ireland; 8 College of Life Sciences, University of Leicester, Leicester, United Kingdom; University of Mississippi Medical Center, UNITED STATES

## Abstract

Preeclampsia remains a leading cause of maternal and perinatal morbidity and mortality. Accurate prediction of preeclampsia risk would enable more effective, risk-based prenatal care pathways. Current risk assessment algorithms depend on clinical risk factors largely unavailable for first-time pregnant women. Delivering accurate preeclampsia risk assessment to this cohort of women, therefore requires for novel biomarkers. Here, we evaluated the relevance of metabolite biomarker candidates for their selection into a prototype rapid, quantitative Liquid Chromatography-tandem Mass Spectrometry (LC-MS/MS) based clinical screening assay. First, a library of targeted LC-MS/MS assays for metabolite biomarker candidates was developed, using a medium-throughput translational metabolomics workflow, to verify biomarker potential in the Screening-for-Pregnancy-Endpoints (SCOPE, European branch) study. A variable pre-selection step was followed by the development of multivariable prediction models for pre-defined clinical use cases, i.e., prediction of preterm preeclampsia risk and of any preeclampsia risk. Within a large set of metabolite biomarker candidates, we confirmed the potential of dilinoleoyl-glycerol and heptadecanoyl-2-hydroxy-sn-glycero-3-phosphocholine to effectively complement Placental Growth Factor, an established preeclampsia biomarker, for the prediction of preeclampsia risk in first-time pregnancies without overt risk factors. These metabolites will be considered for integration in a prototype rapid, quantitative LC-MS/MS assay, and subsequent validation in an independent cohort.

## Introduction

Preeclampsia remains a leading cause of maternal death throughout the world and is responsible for considerable neonatal morbidity and mortality [1]. Management of preeclampsia risk throughout pregnancy therefore remains a key aspect of prenatal care worldwide. The accurate prediction of preeclampsia risk early in pregnancy has been flagged as a research priority in many clinical guidelines [2, 3] as it would enable risk-based prenatal care pathways, and hence more effective utilisation of prenatal care resources. The possible patient benefits of accurate preeclampsia risk prediction have recently been reaffirmed in the ASPRE trial, which reported that aspirin prophylaxis in women identified to be at risk of preeclampsia before 37 weeks of gestation, so-called preterm preeclampsia, reduced the incidence rate of preterm preeclampsia by 62% [4].

The National Institute for Health and Clinical Excellence (NICE) in England and the American College of Obstetricians and Gynecologists (ACOG) in the United States recommend the use of maternal demographic, obstetrical history and overt medical risk factors in simple dichotomous classifications to determine low or high risk status for the development of preeclampsia [5, 6]. However, the prediction accuracy of such approaches is limited. Using the NICE guidelines, sensitivity of 39% for preterm preeclampsia and 34% for term preeclampsia at 10.3% false positive rate (FPR) were reported; the corresponding sensitivity using the ACOG recommendations were 90% and 89%, but at 64.3% FPR [5, 7, 8]. This led clinical researchers to develop multivariable models which utilize these clinical risk factors to compute risk scores, as recently reviewed by Brunelli et al [9]. Several of these were subjected to external validation in two recent Dutch studies [10, 11], showing such models do have some moderate risk prediction ability, with some likely to outperform clinical guidelines [10]. However, these studies also re-iterated that the predictive performance decreased significantly in first-time pregnant women. Myers et al. further corroborated this finding by showing that in a population of low risk nulliparous women, detection rates for preterm preeclampsia were only 16.1%, 26.5% and 22.2% for respectively the NICE classifier [5], the maternal risk based competing risk model underpinning the classifier used in the Combined Multimarker Screening and Randomized Patient Treatment with Aspirin for Evidence-Based Preeclampsia Prevention (ASPRE) trial [12], and an alternative risk scoring method as proposed by Sovio et al [13, 14].

The need for novel biomarkers to improve preeclampsia prediction in nulliparous women without apparent risk factor is further emphasized by the fact that nulliparity is associated with increased risk (RR = 2.1 (1.9 to 2.4)) and accounts for the largest single population attributable fraction (32.3%) for preeclampsia [15].

To date, the most studied biomarker in preeclampsia is Placental Growth Factor (PlGF), the levels of which tend to drop in a sub-group of women destined to develop preeclampsia [16], and which has some demonstrated risk prediction potential [17]. Its use, in combination with maternal characteristics, medical history, obstetric history, blood pressure and, where available, uterine artery doppler measurements, is gaining acceptance for prediction of preterm preeclampsia risk in the general pregnancy population [18]. Yet additional biomarkers are still required to complement PlGF to improve preeclampsia risk prediction in nulliparous women without apparent risk factors.

The aim of this work was to inform the development of a novel biomarker-assisted test for the early and accurate prediction of preeclampsia risk in low-risk nulliparous women. With preeclampsia recognised as being a syndromic condition, accurate prediction of preeclampsia will require for several biomarkers to account for different preeclampsia subtypes and/or patient risk profiles [19–21]. Because liquid chromatography-triple quadrupole mass spectrometry (LC-QqQ-MS) is a well-established analytic technique used in clinical laboratories worldwide for multiplex metabolite analysis, such as neonatal screening for inborn errors of metabolism [22], we set metabolite “assay-ability” by LC-QqQ-MS as a stage-gate biomarker selection criterion. This will ensure that any biomarker panel identified will be easily portable to *in vitro* diagnostic workflows already operated in clinical laboratories.

We previously reported a metabolomic biomarker discovery study for early pregnancy prediction of later preeclampsia [23]. Building on these results (and those of others; S1 File & S1 Table), we set out to consolidate a panel of metabolite biomarkers which could be used in conjunction with PlGF to deliver accurate preeclampsia prediction in nulliparous women.

Firstly we developed a translational research workflow based on LC-QqQ-MS based metabolite quantification; only candidate biomarkers amenable to this workflow were considered for further analysis in a case-control study. Biomarkers with univariable prediction merits were pre-selected for the development of risk predictor models. Models were evaluated in function of two pre-defined clinical use scenarios: (1) identify women at risk of developing preterm preeclampsia (primary objective), and (2) identify women at risk of developing preeclampsia at any stage of the pregnancy (secondary objective). We found that combining dilinoleoyl-glycerol (DLG) with PlGF effectively predicted increased preterm preeclampsia risk at ca. 15 weeks of gestation. The further addition of heptadecanoyl-2-hydroxy-sn-glycero-3-phosphocholine (1-HGP) expanded the capacity to also identify pregnant women at decreased risk of developing any form of preeclampsia. PlGF, DLG and 1-HGP were therefore proposed for progression to the final development stage of a risk prediction test for (preterm) preeclampsia and subsequent validation in the IMproved Pregnancy Outcomes by Early Detection (IMPROvED) study [24].

## Materials and methods

### Study design

Previously reported metabolomic biomarkers discovered in plasma samples from participants of the New Zealand and Australian Cohort of the Screening for Pregnancy Endpoints (SCOPE) study were considered [23]. A curation was performed to address any metabolite identification uncertainty, followed by a pragmatic purging of metabolites without readily available reference materials. The resulting metabolite candidates were selected for LC-MS/MS assay development. Upon establishment of the Analytical Workflow (see below), the selection was supplemented with some additional putative metabolite biomarkers as reported by others; plausibility, ready availability of reference materials, and workflow compatibility were considered (S1 File & S1 Table). The resulting panel of putative metabolite biomarkers was then further assessed for its potential to meet pre-defined clinical use cases in a case-control study (see below).

### Study population

All samples were obtained from participants in the European centres of the SCOPE study (Cork, Ireland; Leeds, London and Manchester, UK) between November 2004 and August 2008. Blood samples were prospectively collected from low-risk nulliparous women between 14 weeks and zero days and 16 weeks and six days gestation (or 15±1 week). The diagnosis of preeclampsia was made at any stage during pregnancy after recruitment until delivery, or in the first 2 weeks postpartum.

Preeclampsia was defined according to the International Society for the study of Hypertension in Pregnancy (ISSHP) [25] (S2 File). Clinical data on known preeclampsia risk factors were collected at 15±1 and 20±1 weeks of gestation by interview and examination of the women. Ultrasound data were obtained at 20 weeks on fetal measurements, anatomy, uterine and umbilical artery Doppler, and cervical length. Fetal growth, uterine and umbilical Dopplers were measured at 24 weeks. Pregnancy outcome was tracked and each woman seen within 48 hours of delivery at which stage new-born measurements were obtained [26]. Customised birthweight centiles were based on the UK customised centiles, adjustments were made for maternal height, weight at 15±1 weeks visit, ethnicity, sex and weight of the new-born and gestation at delivery [27].

#### Inclusion and exclusion criteria

The inclusion criteria were nulliparity, singleton pregnancy, gestational age between 14 weeks and zero days and 16 weeks and six days, and informed consent to participate. The exclusion criteria applied included: *i*) uncertainty of last menstrual period (LMP) and unwillingness to have an ultrasound scan at ≤ 20 weeks’ gestation; *ii*) known major fetal anomaly or abnormal karyotype; *iii*) underlying medical conditions (essential hypertension treated pre-pregnancy, moderate-severe hypertension at booking [≥160/100 mmHg], diabetes, renal disease, systemic lupus erythematosus, anti-phospholipid syndrome, sickle cell disease, HIV positive), previous knife cone biopsy, ≥3 miscarriages or ≥3 terminations, current ruptured membranes, major uterine anomaly, cervical suture; and i*v*) treatment with: long term steroids, low-dose aspirin, calcium (>1 g/24h), eicosopentaenoic acid, vitamin C ≥1000 mg, vitamin E ≥ 400 IU, or low molecular weight heparin.

#### Ethical considerations

Ethics approval was gained from local ethics committees of each participating centre (Manchester, Leeds, and London 06/MRE01/98, Cork ECM5 (10) 05/02/08) and written informed consent was obtained from all participants. Collection of data and biological samples complied with standardised procedures in all participating centres and was conducted in accordance with the principles of the Declaration of Helsinki.

#### Case-control study

Biobanked EDTA plasma samples from all participants with a confirmed pregnancy outcome of the European branch of SCOPE were available. Within the n = 2364 cohort of pregnant women, 97 developed preeclampsia and 2266 did not develop preeclampsia. EDTA plasma samples from the 97 preeclampsia case patients and 335 randomly selected non-preeclampsia control patients were considered for analysis in the case-control study. The demographic and clinical characteristics of the control population selected for the study were compared pairwise and the lack of significant bias was verified (p<0.01, multiple testing correction, Chi square, Mann Whitney U test, Kruskal-Wallis test and Spearman correlation test as applicable).

### Clinical use cases

In consultation with a panel of clinicians, two relevant clinical use scenarios were established for stratifying nulliparous pregnant women without overt risk factors to their preeclampsia risk. These translated to three performance criteria for eventual predictor models relevant to the intended use population: (1) a *rule-in* classification to identify women at high risk of developing preterm preeclampsia (risk ≥ 5%, Positive Predictive Value (PPV) ≥0.05); (2a) a *rule-in* classification identifying women at high risk of developing preeclampsia at any stage of the pregnancy (risk ≥15%, PPV≥0,15); and (2b) a *rule-out* classification identifying women at low risk of developing preeclampsia at any stage of the pregnancy (risk ≤1%, Negative Predictive Value (NPV) ≥0.99). Given that the target pregnancy population constitutes healthy first-time pregnant women, the comparator for the different classifications was defined as prediction models constituting any of the following established predictors: PlGF, body mass index (BMI) and Mean Arterial Pressure (MAP): the second set of blood pressure measurements was used to calculate MAP, as reported earlier [28].

### Reagents and instrumentation

Chemicals and reagents used were of high-performance liquid chromatography (HPLC) and mass spectrometry (MS) grade or higher (Fischer Scientific, Blanchardstown, Ireland). Metabolite reference substances and stable isotope-labelled standards were purchased from Fluka (Arklow, Ireland), Fischer Scientific (Blanchardstown, Ireland), IsoSciences (King of Prussia, PA, USA), Sigma-Aldrich (Wicklow, Ireland), Avanti Lipids (Alabaster, Alabama, USA), QMX Laboratories (Thaxted, UK), LGC (Teddington, U.K), Alfa Chemistry (Holtsville, NY, USA), Generon (Maidenhead, UK), Larodan (Solna, Sweden) and R&D Systems (Abingdon, UK).

For sample preparation, a liquid handling robot, equipped with a 96 LT disposable Tip Head, an orbital shaker station and a Peltier Thermal Station, was used (Agilent Bravo Automated Liquid Handling Platform, Agilent Technologies, Santa Clara, CA, USA).

Targeted metabolite LC-MS/MS analysis was performed using a 1260 Infinity LC system (Agilent Technologies, Waldbronn, Germany) coupled to an Agilent Triple Quadrupole 6460 mass spectrometer (QqQ-MS) equipped with a JetStream Electrospray Ionisation (ESI) source (Agilent Technologies, Santa Clara, CA, USA).

### Analytical workflow

An analytical workflow which complied with pre-defined requirements (outlined in S3 File) was developed. A library of targeted LC-MS/MS assays, based on Multiple Reaction Monitoring (MRM), was established for the pre-selected metabolites and, if commercially available, corresponding stable-isotope labelled metabolite internal standards (SIL-IS). The panel of metabolites and SIL-IS were distributed over two multi-analyte LC-MS/MS methods. These featured two complementary chromatographic separations, i.e., Reversed Phase Liquid Chromatography (RPLC) and Hydrophilic Interaction Liquid Chromatography (HILIC). For RPLC, Zorbax Eclipse Plus C18 Rapid Resolution HD 2.1mm x 50mm, 1.8-Micron (P.N. 959757, Agilent Technologies) columns were used, and for HILIC, Ascentis Express HILIC 15cm x 2.1mm, 2.7 Micron (P.N. 53946-U, Sigma-Aldrich) columns. Both chromatographic methods used 10-minute gradient elution programs and solvent systems enabling direct hyphenation with ElectroSpray Ionisation (ESI)-MS detection. ESI polarity switching was applied in function of metabolite ionisation characteristics. Mass spectrometry instrument parameters were optimised, and LC-MRM assay parameters established for all metabolites and SIL-IS. Further details on the assays and LC-MRM parameters are available in S4 File and S2, S3 Tables respectively.

### Sample analysis

Clinical samples (40 μL), calibrators and Quality Control samples (QCs) were re-configured in analytical batches of 96 samples. The positions of Calibrators (n = 8), Analytical QCs (2*3), and study pool QCs (n = 9) were kept identical across batches. Fifteen percent of clinical samples were selected for duplicate analysis. The samples, inclusive the duplicate samples, were randomised for the analytical process using stratified randomisation over clinical centre, ethnicity, and preeclampsia. The absence of potential sources of experimentally induced bias was confirmed by computing pairwise dependencies between the randomisation outputs (selection, batch, order) and clinical parameters; no significant associations were found. Further information on the study and sample preparation is given in S5 File and S4, S5 Tables.

All batches were first analysed by RPLC-MS/MS and then by HILIC-MS/MS. Following mass spectrometric analysis, the mass spectrometric signals of all measured quantifier and qualifier transitions of the 54 assay-able metabolites and 38 SIL-IS were quantified using a pre-defined quantification method compiled in Masshunter Quant Software vB.07.00 (Agilent Technologies, Santa Clare, CA, USA) across all the samples analysed in the study. All data were then reviewed by two independent analysts and any erroneous signal integration calls made by the software curated; all manual curation was recorded for data integrity purposes.

PlGF was analysed earlier as part of a large-scale assessment of putative protein biomarkers within the SCOPE study using a Luminex Sandwich assay [28]. The latter data were made available by the SCOPE consortium for this study.

### Data pre-processing and quality assurance

All laboratory personnel were blinded to sample status (case or control) at all stages of the study. Structured review of the calibrator signal data and signal data across all sample types was performed to confirm metabolite quantifier transition selection, absence of interference in the SIL-IS transitions, and final metabolite quantification metric. Next, pairwise dependencies between metabolite quantification metrics and recorded experimental variables were computed using the appropriate statistical tests; no aberrant correlations were found other than some minor inter-day batch effects in some of the quantifications. For the metabolite quantifications exhibiting batch-to-batch differences, the relative concentrations were scaled per batch using the ratio of the median concentration for the given batch over the overall median concentration (S6 Table).

Data missingness and imprecision criteria were assessed: data missingness for a given metabolite quantification should be < 20% across all clinical samples, with the exception of cotinine, a reporter metabolite for smoking status. Two imprecision metrics, expressed as coefficient of variance (CV), were calculated: 1) for the replicate study-QC pool samples across all batches and 2) for the 15% technical duplicates (randomly distributed across all samples). Metabolites were selected when %CV ≤ 25% for both metrics. Summary of the assay selection for biomarker analyses based on quality assurance evaluation is provided in S6 Table.

### Statistical analysis

Statistical analyses were performed using the statistical software R version 3.5 [29].

#### Predictor selection

In a first phase, variables were evaluated based on their stand-alone predictive performance for preterm preeclampsia (<37 weeks of gestation), term preeclampsia (> = 37 weeks of gestation) and preeclampsia. Variables considered were: all analytes as well as the established risk factors for preeclampsia, blood pressure (MAP) and BMI. The discriminative performance of each individual variable was quantified using the area under the receiver operating curve (AUROC) [30]. Variables with a lower limit of the 95% confidence interval of AUROC greater or equal to 0.50 (p≤0.05) and AUROC greater than 0.60 were selected as potential predictive markers for preeclampsia. In addition, false discovery rates (FDR) were evaluated across the outcomes using label permutation as described by Storey and Tibshirani using a conservative value for the proportion of truly null values, π_0_, of 1 and 2 x 10^4^ permutations [31].

#### Modelling

The concentrations and relative concentrations of analytes were log-transformed before any modelling. Analysis was performed on complete cases.

First, multivariable models were developed for the outcome ‘any preeclampsia’ using partial least squares–discriminant analysis (PLS-DA) [32]. Comparator models based on any combination of PlGF, MAP and BMI were generated and evaluated in view of the clinical use cases defined (S7 Table). Then, PLS-DA models for all preeclampsia were generated for all combinations of PlGF, DLG and 1-HGP, followed by their evaluation. Secondly, recursive partitioning was applied [33, 34] as an approximation for a Bayesian approach. Models were trained for each of the two outcomes with all combinations of one to three of the preselected predictors. Two to three partitions were made. PlGF was imposed as the first partition step. One to two predictors per step were allowed. When two predictors were used in a partition, these were combined using PLS-DA. To bias these PLS-DA models on non-placental preeclampsia, they were trained on patients with high PlGF, defined as a PlGF concentration higher or equal to the concentration corresponding to the observed maximum accuracy for the prediction of any preeclampsia. Again, comparator models using PlGF, MAP and BMI were generated (Table 3 and S7 Table) as well as partitioning models based on combinations of PlGF, DLG and 1-HGP.

All models were evaluated for the clinical use cases defined. For all models, cut-off values for concentrations and scores were selected based on the rule-in PPV and rule-out NPV criteria. PPV, NPV, sensitivity and specificity, and their respective 95% confidence intervals were estimated at the given cut-offs using bootstrapping. For each model, the superiority of the rule-in sensitivity and rule-out superiority as compared to reference models was computed by estimating a difference in sensitivity or specificity using bootstrapping and paired T test (2,000 replicates, one-tailed test, Bonferroni correction for multiple testing, p<0.05). The reference models were the single marker PlGF, the linear model PlGF+DLG and the recursive partitioning models PlGF||DLG and PlGF||MAP||BMI respectively.

## Results

### Study population

A nested case-control study (n = 432) was performed within the 2364 women recruited into the European SCOPE cohort, for whom pregnancy outcomes were known. Twenty-three women developed preterm preeclampsia (1%) and seventy-four (4%) developed term preeclampsia. Background characteristics and outcomes of pregnancy in women who did and did not develop preeclampsia are shown in Table 1. Mean age, and gestational age at sampling did not differ significantly among the groups. Significant differences are observed for BMI and blood pressure at sampling. Gestational age at delivery was lower in preeclampsia cases compared with controls (no preeclampsia). Likewise, the babies born to preeclamptic women were significantly lighter than the babies in the control group.

**Table 1:** Baseline characteristics of the study population.

	No Preeclampsia		Preeclampsia (n = 97)
	SCOPE study (n = 2266)	Samples selected (n = 335)	
**Gestation age at sampling, wks**	15.5 (0.733)	15.6 (0.726)	15.6 (0.698)
**Maternal age, yrs**	30 (27–33)	30 (27–33)	30 (27–34)
**White ethnicity**	2145 (95%)	315 (94%)	92 (95%)
**BMI at 15 weeks, kg/m2**	23.9 (21.9–26.8)	23.5 (21.9–26.8)	26.1 (23.1–29.4) *
**Waist circumference at 15 wks, cm**	80 (74–86)	80 (74–86)	84 (78–91) *
**Smoker at 15 wks**	234 (10%)	42 (13%)	8 (8.2%)
**Smoker during first trimester**	617 (27%)	106 (32%)	28 (29%)
**DBP at 15 wks, mm Hg**	66 (7.4)	64.8 (7.15)	69.2 (7.31) *
**SBP at 15 wks, mm Hg**	105 (10.3)	104 (10.7)	111 (10.9) *
**MAP at 15 wks, mm Hg**	79.2 (7.65)	78.6 (7.45)	83.1 (7.84) *
**Glucose at 15 wks, mmol/L**	5.1 (4.6–5.8)	5.1 (4.6–5.6)	5.2 (4.5–5.7)
**Max DBP, mm Hg**	80 (74–86)	80 (74–85)	100 (95–107) *
**Max SBP, mm Hg**	126 (119–136)	125 (118–134)	154 (147–170) *
**Proteinuria at delivery**	52 (2.3%)	6 (1.8%)	90 (93%) *
**Gestation age at delivery, wks**	40.3 (39.3–41.1)	40.4 (39.6–41.3)	38.9 (37.1–40.3) *
**Preterm delivery**	97 (4.3%)	15 (4.5%)	23 (24%) *
**Customised birthweight centile**	45.2 (22.2–73.1)	44.4 (20.1–72.2)	28.4 (7.9–61.1) *

wks = weeks; yrs = years; DBP = Diastolic blood pressure; MAP = Mean arterial pressure; SBP = Systolic blood pressure. Count, median (interquartile range), mean (standard deviation)

* = p<0.001; Chi square test, Mann Whitney U test or T test as appropriate.

### Metabolite selection

Initially, 58 previously identified putative metabolite biomarkers were selected as inputs for this translational research; metabolites without ready-accessible reference materials available were excluded, except for sphinganine-1-phosphate for which assay parameters could be inferred from sphingosine-1-phosphate, leaving fifty-two metabolites for assay development. An additional set of 20 putative metabolite biomarkers from other sources (S1 Table), deemed compatible with the analytical workflow and with reference materials available, were chosen for assay development. Eighteen metabolites were lost as they were found to be “non-assayable” with the analytical workflow developed for this translational work.

From the fifty-four (54) metabolites analysed in the case-control study, forty-two complied with the pre-set quality assurance criteria. Two exceptions were allowed, 25-Hydroxyvitamin D3 and Eicosapentaenoic acid, which missed the criterion in one of the two types of QC samples considered. One assay was removed due to being non-specific: the signal constituted contributions of 2-methylglutaric acid, glutamine and an unknown metabolite. Also, one opportunistic marker was assessed: there were no detectable levels for 1,2-Dioctanoyl-sn-glycero-3-phosphocholine, yet the same assay delivered a consistent signal at a slightly different retention time. Comparison with reference materials confirmed this compound to be 1-heptadecanoyl-glycero-3-phosphocholine, the latter was therefore analysed. This resulted in data for forty-three metabolites being progressed to biomarker evaluation (S6 Table).

### Predictor selection

Biomarkers and biometric variables selected based on their stand-alone prediction performance of preterm, term or any preeclampsia are shown in Table 2. Mean Arterial Pressure (MAP) showed significant discriminative performance for term and all preeclampsia (AUROC = 0.67 and 0.69 respectively). BMI delivered similar discrimination for preterm, term and all preeclampsia (AUROC = 0.63 and 0.65 respectively). DLG and PlGF stand-alone prediction performances were higher for preterm preeclampsia (AUROC = 0.70 and 0.73 respectively). 1-HGP showed similar discriminative performance for both preterm and term preeclampsia (AUC = 0.61) but this performance was only significantly superior to 0.50 for term preeclampsia (p<0.05). With high FDR associated with the predictor selections for preterm preeclampsia and term preeclampsia, only the predictors as selected for preeclampsia were considered as variables for modelling.

**Table 2:** Selection of predictors for multivariable modelling.

Selected predictors	Preterm Preeclampsia; FDR: 59%		Term Preeclampsia; FDR: 7.4%		Preeclampsia; FDR: 2.8%	
	AUROC (95%CI)	Effect size (95%CI)	AUROC (95%CI)	Effect size (95%CI)	AUROC (95%CI)	Effect size (95%CI)
**MAP**	N/P	N/A	0.69 (0.63–0.76)	↑ 5.3 mmHg (3.3–7.7)	0.67 (0.61–0.73)	↑ 4.7 mmHg (2.7–6.7)
**BMI**	0.65 (0.54–0.75)	↑ 1.9 kg/m^2^ (0.4–3.5)	0.63 (0.56–0.70)	↑ 2.0 kg/m^2^ (0.9–3.1)	0.64 (0.57–0.70)	↑ 2.0 kg/m^2^ (1.0–2.9)
**DLG**	0.70 (0.59–0.82)	↑ 1.45 (1.18–1.78)	N/P	N/A	0.61 (0.55–0.67)	↑ 1.23 (1.09–1.37)
**Choline**	0.61 (0.50–0.72)	↑ 1.09 (0.99–1.20)	N/P	N/A	N/P	N/A
**Isoleucine**	N/P	N/A	0.61 (0.54–0.68)	↑ 1.12 (1.04–1.20)	N/P	N/A
**1-HGP**	N/P	N/A	0.61 (0.53–0.68)	↓ 0.89 (0.82–0.96)	0.61 (0.54–0.67)	↓ 0.89 (0.83–0.95)
**2-Hydroxybutanoic acid**	0.62 (0.50–0.73)	↑ 1.16 (0.99–1.37)	N/P	N/A	N/P	N/A
**NG-monomethyl-L-arginine**	0.61 (0.50–0.72)	↑ 1.08 (0.99–1.18)	N/P	N/A	N/P	N/A
**Decanoylcarnitine**	N/P	N/A	0.60 (0.53–0.67)	↑ 1.32 (1.08–1.63)	N/P	N/A
**PlGF**	0.73 (061–0.85)	↓ 0.43 (0.29–0.89)	N/P	N/A	0.60 (0.53–0.67)	↓ 0.71 (0.57–0.89)

Predictor selection based on predictive performance as a single marker (AUROC >0.60, LCI ≥0.50) and median effect size* compared to controls.

*Effect size: median difference for BMI and MAP and median fold change for analyte concentration (95% confidence interval); AUROC = area under the receiver operating curve (95% confidence interval); LCI = lower confident interval; CI = confident interval; N/P = No predictive performance; N/A = Not applicable; MAP = Mean Arterial Pressure; BMI = Body mass index; DLG = Dilinoleoyl-glycerol; 1-HGP = 1-Heptadecanoyl-2-hydroxy-sn-glycero-3 phosphocholine

↑ = up-regulated compared to controls

↓ = down-regulated compared to controls; FDR = false discovery rate.

### Modelling

**Fig 1:**
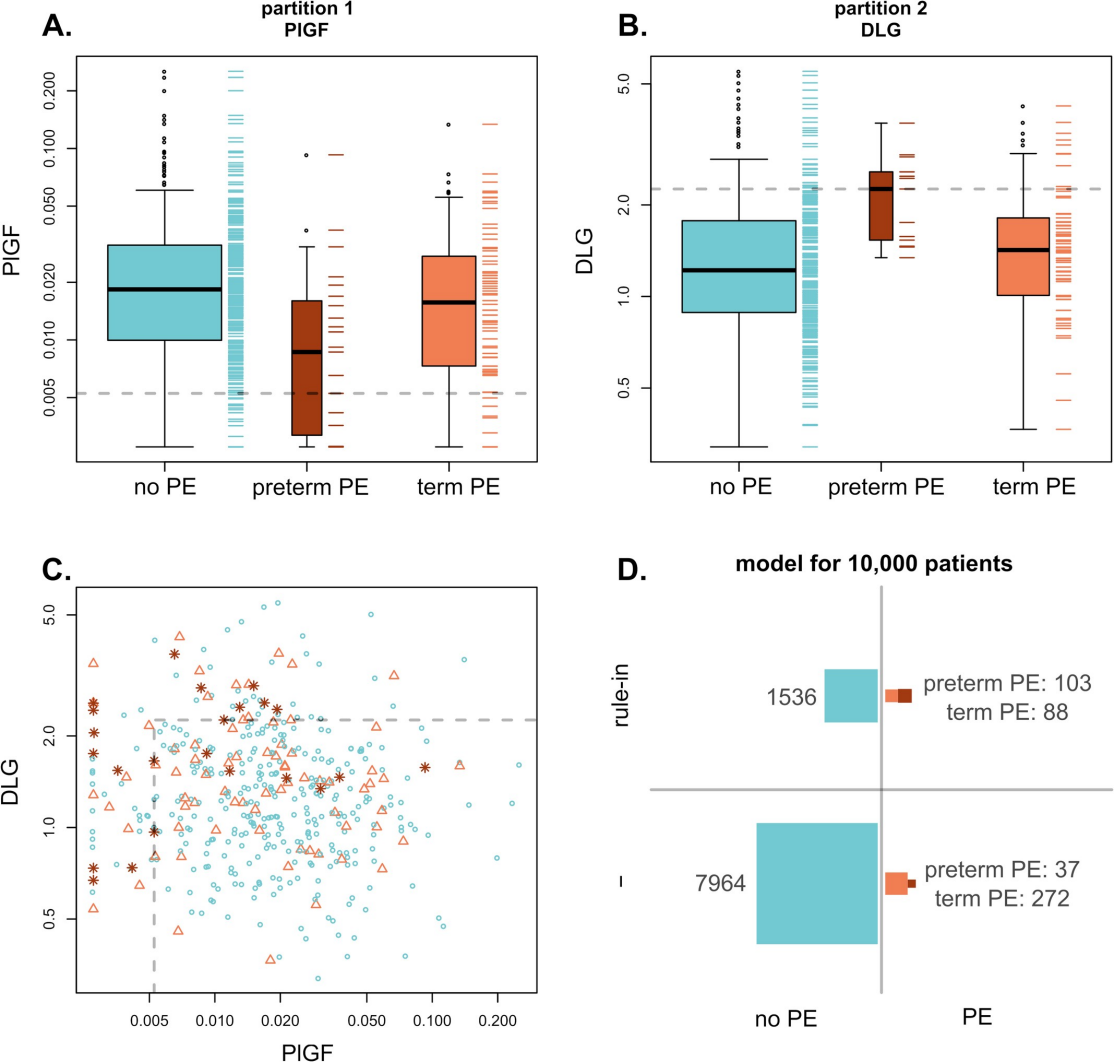
Partitioning rule-in model (PlGF || DLG). (A) Partition 1: PlGF concentration (ng/mL) versus clinical outcome PlGF cut-off value = 0.00526 (dotted line). (B) Partition 2: DLG relative concentration versus clinical outcome for patients ruled-out in partition 1. DLG cut-off value = 2.26 (dotted line). (C) Partitions 1 and 2: DLG relative concentration versus PlGF concentration (red stars: preterm preeclampsia, orange triangles: term preeclampsia, blue circles: no preeclampsia). Dotted line: DLG and PlGF cut-off values. (D) Estimated predictive performance of the model for 10,000 patients.

Model development was targeted to answer two pre-set outcome based clinical use scenarios (preterm preeclampsia and any preeclampsia), and three associated minimal performance requirements, expressed as Positive and Negative Predictive Value thresholds (PPV and NPV, as detailed in Methods–Clinical use cases). The multivariable models developed within this framework are shown in Table 3; the comparator models based on the classic predictors MAP, BMI and PlGF are available in S7 Table. For the primary objective, i.e., the detection of women at increased risk for preterm preeclampsia at a pre-set PPV cut-off of ≥ 0.05, the observed sensitivity using only PlGF was 48% (PPV = 0.05), and up to 61% for the comparator models combining PlGF with MAP and/or BMI. Combining PlGF with DLG by means of recursive partitioning increased the sensitivity significantly to 74% (PPV = 0.06). The resulting partitioning rule-in model (PlGF || DLG) is shown in Fig 1. Predictor cut-off values in each partition step were 0.00526 ng/mL and 2.26 relative concentration (RC) for PlGF and DLG respectively. By combining PlGF with a two-predictor partial least squares discriminant analysis (PLS-DA) model constituting DLG and 1-HGP (PlGF || (DLG+1-HGP)), the sensitivity could be increased further to 78% (PPV = 0.05; S1 Fig).

**Table 3:** Prediction models for the pre-set clinical use scenarios.

**Primary Objective: Prediction of increased risk of preterm preeclampsia at PPV > = 0.05**						
*Model*	*Method*	*AUROC*	*PPV*	*Sensitivity*	*Specificity*	
**PlGF (ref)**	N/A	0.73 (0.61–0.85)	0.059 (0.033–0.096)	**0.48** (0.26–0.70)	0.89 (0.86–0.93)	
**DLG**	N/A	0.70 (0.59–0.82)	0.054 (0.027–0.091)	**0.39** (0.22–0.61)	0.90 (0.87–0.93)	
**PlGF + DLG (ref)**	PLS-DA	0.79 (0.69–0.89)	0.056 (0.034–0.082)	0.57* (0.39–0.74)	0.86 (0.82–0.90)	
**PlGF + DLG + 1-HGP**	PLS-DA	0.78 (0.69–0.88)	0.052 (0.035–0.075)	0.65*° (0.43–0.83)	0.83 (0.79–0.87)	
**PlGF || MAP || BMI (ref)**	RP	N/A	0.054 (0.036–0.078)	0.61*° (0.43–0.78)	0.85 (0.81–0.89)	
**PlGF || DLG (ref)**	RP	N/A	0.061 (0.044–0.085)	0.74*°^**§**^ (0.57–0.91)	0.84 (0.80–0.88)	
**PlGF || (DLG + 1-HGP)**	RP & PLS-DA	N/A	0.052 (0.039−0.069)	0.78*°^**†**^^**§**^ (0.61−0.96)	0.80 (0.75−0.84)	
**PlGF || DLG || 1-HGP**	RP	N/A	0.060 (0.043–0.084)	**0.74** *°^**†**^^**§**^ (0.57–0.91)	0.84 (0.79–0.87)	
**Secondary Objective: Prediction of decreased risk of any preeclampsia at NPV> = 0.99**						
***Model***	*Method*	*AUROC*	*NPV*	***Specificity***		*Sensitivity*
**PlGF (ref)**	N/A	0.60 (0.53–0.67)	1.00 (1.00–1.00)	**0.02** (0.01–0.03)		1.00 (1.00–1.00)
**DLG**	N/A	0.61 (0.55–0.67)	1.00 (0.95–1.00)	**0.01** (0.00–1.00)		1.00 (0.00–1.00)
**PlGF + DLG (ref)**	PLS-DA	0.64 (0.57–0.70)	0.99 (0.97–1.00)	0.06* (0.04–0.09)		0.99 (0.97–1.00)
**PlGF + DLG + 1-HGP**	PLS-DA	0.66 (0.60–0.72)	1.00 (1.00–1.00)	**0.03** (0.01–0.04)		1.00 (1.00–1.00)
**PlGF || MAP || BMI (ref)**	RP	N/A	0.99 (0.98–1.00)	0.32*°^**†**^ (0.27–0.37)		0.94 (0.89–0.98)
**PlGF || DLG (ref)**	RP	N/A	0.99 (0.98–1.00)	0.13*° (0.10–0.17)		0.98 (0.95–1.00)
**PlGF || (DLG + 1-HGP)**	RP & PLS-DA	N/A	0.99 (0.98−1.00)	0.22*°^**†**^ (0.18−0.26)		0.96 (0.92−0.99)
**PlGF || DLG || 1-HGP**	RP	N/A	0.99 (0.98–1.00)	0.37*°^**†**^^**§**^ (0.32–0.42)		0.93 (0.88–0.98)

(ref) reference models for superiority testing

* significantly higher compared to PlGF

° significantly higher compared to PlGF+DLG

^†^ significantly higher compared to PlGF||DLG

^§^ significantly higher compared to PlGF||MAP||BMI (T test, p<0.05, Bonferroni multiple testing correction). CI Confidence interval; DLG = Dilinoleoyl-glycerol; 1-HGP = 1-heptadecanoyl-2-hydroxy-sn-glycero-3 phosphocholine; RP = recursive partitioning; PLS-DA = partial least squares discriminant analysis. N/A = not applicable.

With regards to predicting increased risk for any type of preeclampsia at a pre-set PPV cut-off of ≥ 0.15 (secondary objective), comprehensive analysis of the generated prediction models revealed that no classifier compliant with the PPV≥0.15 requirement could be identified.

**Fig 2:**
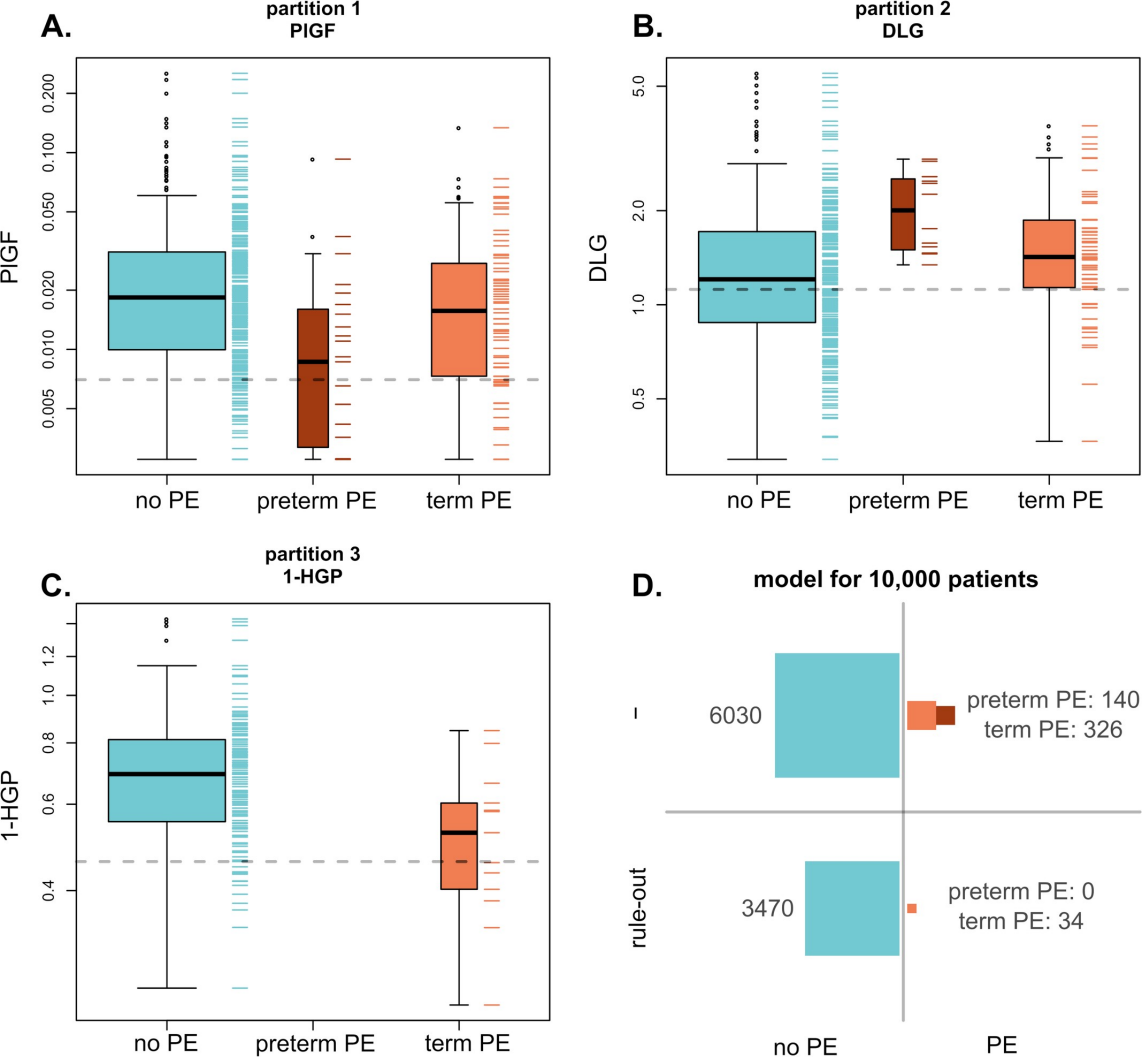
Partitioning rule-out model (PlGF || DLG || 1-HGP). (A) Partition 1: PlGF (ng/mL) versus clinical outcome. PlGF cut-off value = 0.00702 (dotted line). (B) Partition 2: DLG relative concentration vs clinical outcome for patients ruled-out in partition 1. DLG cut-off value = 1.12 (dotted line). (C) Partition 3: 1-HGP relative concentration versus clinical outcome for patients ruled-out in partitions 1 and 2. 1-HGP cut-off value = 0.458 (dotted line). (D) Estimated predictive performance of the model for 10,000 patients.

In models developed to meet the “rule-out” performance criteria (Table 3), specificity at the target NPV≥0.99 for preeclampsia improved from 0.02 (PlGF only) to 0.37 when combining PlGF, DLG and 1-HGP predictors using recursive partitioning. The resulting partitioning rule-out model (PlGF || DLG || 1-HGP) and the estimated predictive performance are shown in Fig 2. Predictor cut-off values in each partition step were 0.00702 ng/mL, 1.12 RC and 0.458 RC for PlGF, DLG and 1-HGP respectively. This model compares favourably to the comparator models combining PlGF with MAP and/or BMI; the best comparator model, i.e., the recursive partitioning model PlGF || BMI || MAP delivers a rule-out specificity of 0.32.

## Discussion

In this study we re-evaluated the relevance of a set of putative metabolite biomarkers to further inform the development of a test to predict preeclampsia in nulliparous women without apparent risk factors. Upon application of both technical assay-ability and data quality assurance criteria, forty-three metabolites, PlGF and the biometric variables MAP and BMI, well-known to associate with preeclampsia, were retained as predictors for consideration.

In line with the findings that placental pathology is more common in preterm preeclampsia [35], we adopted the hypothesis that within the group of women who developed preterm preeclampsia, there would be a greater prevalence of preeclampsia associated with placental insufficiency, whereas the group of women developing term preeclampsia would be more reflective of preeclampsia of maternal origin [36]. Hence, in addition to the outcome preeclampsia, we considered the outcomes preterm and term preeclampsia as two disease subtypes in the process of pre-selecting variables.

This led to an initial selection of twelve predictors, comprising the clinical risk factors relevant in the low-risk population studied (e.g., MAP and BMI), PlGF and nine metabolites from the original set of forty-three quantified metabolites. For all predictors but BMI, the single variables showed discriminative performance which aligned with the prediction of either preterm preeclampsia or term preeclampsia. This observation supports the assumption that in this study population the two disease subtypes considered do indeed align well with distinct underlying physiological pathways. Interestingly, we note that in this study population, MAP is only a stand-alone predictor for term preeclampsia and not for preterm preeclampsia, whereas typically MAP is reported to be a strong stand-alone predictor for preterm preeclampsia [37]. With high FDR associated with the predictors selected for preterm and term preeclampsia, we restricted the final predictor selection for modelling to these predictors which showed performances when assessed for all preeclampsia prediction, i.e., the biometric predictors MAP and BMI and the biomarkers PlGF, DLG and 1-HGP.

Consultation with a panel of clinicians (IMPROvED Consortium meeting, London 2018; see also Acknowledgements) established two relevant clinical use scenarios for stratifying nulliparous pregnant women without overt risk factors to their preeclampsia risk. The primary clinical use scenario was a *rule-in* classification to identify women at high risk of developing preterm preeclampsia. The secondary clinical use scenario was delivering the same preeclampsia risk information to nulliparous pregnant women without overt risk factors, as the risk information available to parous women based on their obstetrics history [38].

In risk stratification, the clinical usefulness of a test is best expressed in terms of positive and negative predictive values [38]. Hence, to establish robust performance targets for the prognostic test(s) for the identified clinical use scenarios, we derived these risk probabilities from published large clinical studies reporting risk probabilities for well-powered population studies. The ASPRE study, involving a cohort of 25,797 pregnancies with mixed prior risk profiles and parity who were screened for preterm preeclampsia, provided a rule-in classification threshold of PPV ≥ 0.05 [39]. The population-based study of Hernandez-Diaz included data from 504,849 parous pregnancies. These authors reported a preeclampsia risk of 14.7% in the second pregnancy for women with a history of preeclampsia, whereas the risk of preeclampsia for parous women without a history of preeclampsia was 1.1% [40].

Considering these “target” inputs led to the establishment of a set of hierarchical goals for the development of predictive models for preeclampsia in nulliparous women without apparent risk factors. The primary goal was defined as the identification of pregnant women at increased risk for preterm preeclampsia with a post-test risk ≥ 0.05 (or PPV ≥ 0.05). Secondary to this, the establishment of generic risk for developing preeclampsia at any stage of pregnancy. For the latter, both the identification of women at high risk, defined as a post-test risk ≥ 15% (PPV ≥ 0.15), and the identification of women at low risk, defined as a post-test risk ≤ 1% (NPV ≥ 0.99), were considered of interest to clinical practice.

Among all preeclampsia biomarkers studied, PlGF is the best understood. Low PlGF levels throughout pregnancy are associated with a disease phenotype which has placental insufficiency as a key characteristic [16, 41–44]. Indeed, it has been shown that low PlGF levels early in pregnancy has some preeclampsia risk prediction merits, especially for preterm preeclampsia wherein placental compromise is more common [16].

Given the multi-factorial nature of preeclampsia, a Bayesian modelling approach may deliver more accurate predictive models than PLS-DA [12, 45]. However, the available number of patients prevented calibration and therefore the use of probabilistic methods. For this reason, recursive partitioning was considered as an alternative as it does not necessitate calibration. In the absence of calibration, generalisability of the models developed cannot be gauged in this study; this will be assessed in a readily available independent cohort [24].

We indeed found that recursive partitioning delivered better prediction than PLS-DA models. By establishing PlGF as the first independent predictor, the recursive partitioning modelling allowed us to align the model development with the pre-defined classification objectives. We found that complementing PlGF with a single metabolite biomarker (DLG) increased the sensitivity of the test from 48% (at PPV = 0.05) to 74% (at PPV = 0.06) for predicting preterm preeclampsia risk in nulliparous women without overt risk factors. Preliminary results show combining 1-HGP with DLG in the second partition may further improve the prediction of preterm preeclampsia, increasing the sensitivity to 78%.

Developing recursive partitioning models with three splits suggested that 1-HGP may also be a third independent predictor. Adding 1-HGP enabled identification of significant fraction of women at low risk of developing preeclampsia (specificity = 36%) in accordance with the pre-set rule-out target of NPV = 0.99.

In this data set we were unable to establish a prediction model which also complied with the other secondary goal: the identification of a sizeable group of women with a post-test risk ≥ 15% of developing any type of preeclampsia in their pregnancy. This may be explained in the context of the competing risk model concept as established for preeclampsia by Wright *et al*. In this model, pregnant women at risk of developing preeclampsia may experience an uncomplicated pregnancy, as their pregnancy (naturally) concluded prior to their preeclampsia risk coming to expression [46].

It is however noted that the list of putative biomarkers considered here was primarily informed by our earlier work [23] and from findings other groups around the same time (S1 Table). Additional putative metabolite biomarkers are still being identified in de-novo metabolomics biomarker studies within other pregnancy demographics [47–50]. Evaluation of these metabolites within the technical and clinical use framework established here, may lead to further prediction improvements and achieving the secondary goal in full.

A review of the literature reveals that DLG and 1-HGP may map onto different but complementary pathways relative to PlGF. Genetic studies have provided new insights into the mechanisms that link intracellular diacylglycerol concentrations to insulin resistance [51, 52], which associates with preeclampsia risk [53]. Erion & Shulman reported that increases in intracellular diacylglycerol content could lead to activation of new protein kinase C which in turn, inhibits insulin action in liver and skeletal muscle [54]. Diacylglycerols in plasma were reported as the basis of a biomarker profile of metabolic syndrome onset in rhesus monkeys; diacylglycerol-derived linoleate levels were markedly upregulated in insulin-resistant metabolic syndrome animals [55]. The physiological metabolic alterations occurring in normal pregnancy: transient excursion into insulin resistance, hyperlipidemia, and increased coagulation are essentially the hallmarks of metabolic syndrome, and are exacerbated in preeclampsia [56, 57]. With the metabolic syndrome manifesting in women with preeclampsia typically associated with insulin resistance and endothelial dysfunction [58], it is therefore plausible that DLG levels reflect risk pathway for the development of preeclampsia that is associated with a pregnancy-specific disposition for metabolic syndrome [56]. With reference to 1-HGP, previous studies have shown that lysophosphatidic acid (LPA), the hydrolysed form of 1-HGP, is a potent mediator of the immune response and could contribute to improper immune activation upregulating the production of inflammatory cytokines [59, 60]. Studies in humans and animal models have indicated that the development of hypertension in preeclampsia has a strong pro-inflammatory component and that placental ischemia is the stimulus for this immune activation [61–64]. In this context, 1-HGP may be related to vascular inflammation and dysfunction. Recently, Fujii et al. reported that LPA signalling may be directly involved in placental homeostasis. They found that dysregulation of LPA signalling as mediated by LPA specific G-protein-coupled receptors (LPAR), and more specifically LPAR3, may be associated with placental dysfunction in preeclampsia [65]. Circulating 1-HGP levels may therefore be indicate LPA signalling issues which in turn could be directly or indirectly be associated with the risk of developing preeclampsia.

Based on the data generated in this study, DLG and 1-HGP are selected as the key markers for the development of a dedicated, rapid, quantitative LC-MS/MS assay prototype.

## Conclusions

In this study, we adopted an end-user focused translational research methodology for the prioritization of putative metabolite biomarkers for further development into a clinical test for preeclampsia risk prediction. Three key determinants were considered in the selection process: compatibility with LC-MS/MS technology already available in clinical laboratories, ability to improve the prediction of the established preeclampsia biomarker PlGF, and ability to meet patient stratification requirements as identified by clinical practitioners.

Using this translational research framework to re-evaluate fifty-eight metabolite biomarker candidates, we were able to prioritise two metabolite biomarkers from an initial pre-selection of nine metabolites, for development of a clinical assay. We found that the metabolites DLG and 1-HGP effectively complemented PlGF in a prediction model for (preterm) preeclampsia in nulliparous women without over risk factors. A review of the literature supports that PlGF, DLG and 1-HGP may represent separate pathways for preeclampsia risk in the population studied.

A strength of this study lies in the well-defined pregnancy risk profile within the SCOPE study, allowing us to focus this translation biomarker research to the pregnancy population most in need for biomarker-assisted risk prediction. In addition, a rational approach considering both technical usability and clinical utility criteria for refining the selection of previously identified putative biomarkers of interest for further test development, was adopted to increase the chance of successfully porting novel biomarkers into clinical practice.

A limitation of this work lies within the ethnic make-up of the SCOPE-Europe study; most of the women were of white ethnicity. In line with the hypothesis that different pregnancy populations may constitute different patient risk profiles, there may be a need for additional biomarkers, representing additional preeclampsia risk pathways, to improve prediction for other ethnicities. Another limitation of this study is the gestational age considered; risk prediction involved samples taken early in the second trimester of pregnancy (15 +/- 1 week of gestation), whereas there is trend to consolidate prenatal risk screening at the end of the first trimester. However, we note that none of the verified metabolite biomarkers show any dependency on gestational age of sampling (S2 Fig).

The next steps in our test development work will involve developing a multiplex clinical assay for the selected markers, as well as further assessment of their generalisability to complement PlGF for the prediction in preeclampsia in other pregnancy populations. In a first instance, the markers will be assessed in IMPROvED; a pan-European multi-centre phase 2A clinical study which recruited ca 4000 low risk primiparous women for the purposes of assessing and refining innovative biomarker-assisted prototype predictive tests [24].

## Supporting information

S1 File Metabolite input selection process. (DOCX)

S2 File Preeclampsia definition. (DOCX)

S3 File Analytical workflow requirements. (DOCX)

S4 File Description LC-MS/MS assays. (DOCX)

S5 File Experimental details of case-control study. (DOCX)

S1 Table Primary metabolite inputs. (DOCX)

S2 Table LC-MRM parameters and instrument specific ionization source settings for the hydrophobic metabolites and associated SIL-IS. (DOCX)

S3 Table LC-MRM parameters and instrument specific ionization source settings for the hydrophilic metabolites and associated SIL-IS. (DOCX)

S4 Table Composition and concentrations of fortification spike mixture. (DOCX)

S5 Table Composition and concentrations of SIL-IS mixture. (DOCX)

S6 Table Quantification metric selection and assay selection for biomarker analyses. (DOCX)

S7 Table Comparator predictors for the pre-set clinical use scenarios. (DOCX)

S1 Fig Partitioning rule-in model (PlGF || DLG + 1-HGP). (DOCX)

S2 Fig Biomarker concentrations in function of gestational age at blood sampling. (DOCX)
