# Supplementary material for: Prediction of preeclampsia risk in first time pregnant women: Metabolite biomarkers for a clinical test
Source: PLoS One. 2020 Dec 28;15(12):e0244369. doi: 10.1371/journal.pone.0244369 (PMC7769282; doi:10.1371/journal.pone.0244369)
Supplement: S1 File — (DOCX) [file pone.0244369.s001.docx]

**S1 File. Metabolite input selection process**

The original research resulted in a robust multivariable predictor [1], yet not all metabolite variables as used in this predictor were unambiguously identified. No assessment of their “fitness” to serve as inputs to a formal (commercial) clinical test development effort was made at the time. Variable attrition from the original predictor was therefore deemed unavoidable when progressing these biomarker findings into a more product-focused translational research framework. For this reason, all the metabolites as reported in Table 2 of the original research were considered as primary metabolite inputs for this translational research effort.

## *Curation of metabolite inputs from primary discovery research*

When the original biomarker discovery research [1] reported metabolite identification ambiguity (e.g. “methylglutaric acid and/or adipic acid”), the different identities were considered separate candidates. In these instances where the original metabolite identification amounted to an identification of a non-discriminative group of metabolites (e.g., “Monosaccharide(s)” or “Vitamin D3 derivatives”), one or more representative metabolites were chosen as an input. (S1 Table; columns one and two)

## *Availability of reference materials*

The availability of reference materials is a basic requirement for the development of targeted LC-MS/MS assays for the metabolites of interest. In addition, the availability of reference materials from (reputable) commercial sources will facilitate any future product manufacturing efforts, as suppliers will be required for provision of materials to e.g. formulate calibrators. (S1 Table; columns three and four).

## *Supplementation of primary metabolite inputs with additional targets*

Where available, reference to relevant literature is made and/or the rationale of selection is given. (S1 Table; second section)

**References**

1. Kenny LC, Broadhurst DI, Dunn W, Brown M, North R a, Mccowan L, et al. Robust early pregnancy prediction of later preeclampsia using metabolomic biomarkers. Hypertension. Oktober 2010;56(4):741–9.
