# Supplementary material for: Prediction of preeclampsia risk in first time pregnant women: Metabolite biomarkers for a clinical test
Source: PLoS One. 2020 Dec 28;15(12):e0244369. doi: 10.1371/journal.pone.0244369 (PMC7769282; doi:10.1371/journal.pone.0244369)
Supplement: S2 File — (DOCX) [file pone.0244369.s002.docx]

# **S2 File. Preeclampsia Definition**

# Preeclampsia was defined according to the International Society for the study of Hypertension in Pregnancy [1] as blood pressure ≥ 140/90 mm Hg after 20 weeks’ gestation (on at least 2 occasions 4 hours apart), the presence of proteinuria (≥ 300 mg/24h (≥ 1g /L), spot urine protein: creatinine ratio ≥30 mg/mmol or urine dipstick protein ≥ 2) and or evidence of multiorgan complications. Multisystem complications included any of the following: i) acute renal insufficiency defined as a new increase in serum creatinine ≥ 100 µmol/L antepartum or >130 µmol/L postpartum; ii) liver disease defined as raised aspartate transaminase and/or alanine transaminase >45 IU/L and/or severe right upper quadrant or epigastric pain or liver rupture; iii) neurological problems defined as eclampsia or imminent eclampsia (severe headache with hyperreflexia and persistent visual disturbance) or cerebral haemorrhage; iv) haematological including thrombocytopenia (platelets <100 x 109/L), disseminated intravascular coagulation or haemolysis, diagnosed by features on blood film (e.g., fragmented cells, helmet cells) and reduced haptoglobin.

**References**

1. Brown MA, Magee LA, Kenny LC, Karumanchi SA, McCarthy FP, Saito S, et al. Hypertensive Disorders of Pregnancy. Hypertension. 2018;72(1):24–43.
