# Supplementary material for: Prediction of preeclampsia risk in first time pregnant women: Metabolite biomarkers for a clinical test
Source: PLoS One. 2020 Dec 28;15(12):e0244369. doi: 10.1371/journal.pone.0244369 (PMC7769282; doi:10.1371/journal.pone.0244369)
Supplement: S3 File — (DOCX) [file pone.0244369.s003.docx]

# **S3 File. Analytical Workflow Requirements**

## *Analytical Detection Technology*

Mass Liquid Chromatography (LC-) directly hyphenated to tandem quadrupole mass spectrometry (QqQ-MS) operated in Multiple Reaction Monitoring (MRM) mode and using with ElectroSpray Ionisation (ESI) was chosen as the analytical detection technology. This type of mass spectrometry, in conjunction with Stable Isotope Dilution [1], is well established in clinical laboratories for the quantitation of small molecules (metabolites, drugs of abuse) in biospecimens. Moreover, MRM, which involves the monitoring of one or more specified mass transitions (precursor/product ion pairs) per analyte, enables development of assay with high specificity [2]. This choice was further informed by the observation that most mass spectrometer manufacturers prioritise listing and/or certifying LC-MS systems based on tandem quadrupole mass spectrometry as medical devices with international regulatory bodies. (US FDA- class I medical device listings, EU – CE-IVD certification).

*Use of biobanked samples*

In view of the rational usage of bio-banked samples, sample volume available for analysis was restricted to ≤50 microliter. Implementation of a sample pre-processing step involving a single freeze-thaw cycle of biobanked specimens to create replicate samples (n≥3) of defined volume and a pooled QC sample [3] was decided upfront.

## *Analytical Determinants*

The analytical workflow had to maximize coverage of the metabolites selected as primary inputs; hence the workflow needed to be relatively invariant to the heterogeneity in physicochemical properties among the selected metabolites. Secondary to this recovery requirement, the simplest sample preparation protocol delivering upon this recovery (in a repeatable way) was to be favored.

Given this research is still translational, the use of relative quantitation was deemed fit-for-purpose based on a cost-benefit assessment weighing the added value of absolute quantitation vs. development cost of establishing absolute quantitation methodology for a wide range of metabolites with limited verified biomarker potential. In relative quantitation, analyte levels were compared across all samples analyzed.

Withstanding no absolute quantification of clinical samples was mandated, relative calibration was to be used to inform assay quality in terms of linearity for the translational research and eventual next test development steps. Additional Quality Assurance requirements were set to include the use of a pooled QC, replicate samples to be distributed over the whole duration of a translational research study, as well as typical QC samples across the linear range (low-medium-high), to enable robust estimation of assay precision (%CV).

## *Preparation Process*

Preparation of samples needed to be compatible with the well-established 96 well format to enable the use of liquid handling robotics as commonly available in clinical laboratories. Based on this, the analytical batch size was decided to correspond the 96 well format; whereby an analytical batch needed to constitute (relative) calibrators, QC samples and clinical samples.

Target Turn Around Time for analyzing a pre-assembled analytical batch was set to be <24h, inclusive of performing system readiness checks and daily LC-MS system qualification.

The process had to enable good study design in terms of enabling facile sample randomization (to avoid experimental bias), monitoring for experimental artefacts and operator blinding.

**References**

1. Pitt JJ. Principles and Applications of Liquid Chromatography- Mass Spectrometry in Clinical Biochemistry. Clin Biochem Rev. 2009;30(February):19–34.

2. CLSI. Liquid Chromatography - Mass Spectromtry Methods; Approved Guideline CLSI document 62-A. October 20. Wayne, PA: Clinical and Laboratory Standards Institute; 2014.

3. Broadhurst D, Goodacre R, Reinke SN, Kuligowski J, Wilson ID, Lewis MR, et al. Guidelines and considerations for the use of system suitability and quality control samples in mass spectrometry assays applied in untargeted clinical metabolomic studies. Metabolomics. 2018;14(6):1–17.
