# Supplementary material for: Prediction of preeclampsia risk in first time pregnant women: Metabolite biomarkers for a clinical test
Source: PLoS One. 2020 Dec 28;15(12):e0244369. doi: 10.1371/journal.pone.0244369 (PMC7769282; doi:10.1371/journal.pone.0244369)
Supplement: S4 File — (DOCX) [file pone.0244369.s004.docx]

# **S4 File. Description LC-MS/MS assays**

Materials and reagents used in the dual separations: LC-MS grade ammonium acetate (NH4OAc) and ammonium formate (NH_4_HCOO) were purchased from Fluka (Arklow, Ireland). LC-MS optima grade acetic acid, acetonitrile (ACN), methanol (MeOH) and 2-Propanol (IPA) were purchased from Fischer scientific (Blanchardstown, Ireland).

## *RPLC-ESI-MS/MS*

The RPLC method was defined by the following settings /parameters; Injection volume: 7 μL; Column oven temperature: 60°C; mobile phase A: H2O:MeOH:200mM NH4OAc at pH 4.5, (92:3:5); mobile phase B: MeOH:ACN:IPA:200mM NH4OAc at pH 45 (35:35:25:5). A linear gradient program was applied: from 10% mobile phase B to 100 %mobile phase B in 10 minutes, using the following gradient – flow rate program:

| **Time (min)** | **%Mobile**  **phase A** | **%Mobile**  **phase B** | **Flow rate (ml/min)** |
| --- | --- | --- | --- |
| 0.00 | 100% | 0% | 0.350 |
| 6.00 | 0% | 100% | 0.5 |
| 8.00 | 0% | 100% | 0.5 |
| 8.10 | 100% | 0% | 0.5 |
| 9.00 | 100% | 0% | 0.5 |
| 10.00 | 100% | 0% | 0.350 |

The efflux of the RPLC column was led directly to the QqQ-MS for mass spectrometric determination of the hydrophobic compounds of interest (see below). For the mass spectrometric method used for analyzing the hydrophobic metabolites of interest, the optimized electrospray ionization source parameters were as follows:

| **Source Parameters** | **Positive mode** | **Negative mode** |
| --- | --- | --- |
| Gas Temperature, °C | 200 | 200 |
| Gas flow, l/min | 13 | 13 |
| Nebuliser, psi | 40 | 40 |
| Sheath Gas Heater | 400 | 400 |
| Sheath Gas Flow | 11 | 11 |
| Capillary, V | 5000 | 3000 |
| V Charging | 300 | 300 |

The specific LC-MRM parameter sets established for the hydrophobic metabolites and associated SIL-IS together with some instrument specific ionization source settings are presented in S2 Table.

## *HILIC-ESI-MS/MS*

The HILIC method was defined by the following settings/parameters; Injection volume: 3 μL, whereby the injection plug was bracketed by 3 μL ACN solvent plugs (injector program); Column oven temperature: 30°C; mobile phase A: 50 mM NH4HCOO (aqueous); mobile phase B: ACN. A linear step gradient program was applied: from 10% mobile phase B to 100 %mobile phase B in 10 minutes. using the following gradient – flow rate program:

| **Time (min)** | **Mobile phase A** | | **Mobile phase B** | **Flow rate (mL/min)** | |
| --- | --- | --- | --- | --- | --- |
| 0.00 | 12% | 88% | | | 0.45 |
| 1.10 | 20% | 80% | | | 0.45 |
| 2.00 | 20% | 80% | | | 0.45 |
| 2.10 | 30% | 70% | | | 0.45 |
| 3.00 | 30% | 70% | | | 0.45 |
| 3.10 | 40% | 60% | | | 0.45 |
| 4.00 | 40% | 60% | | | 0.45 |
| 6.00 | 50% | 50% | | | 0.45 |
| 7.20 | 50% | 50% | | | 0.45 |
| 7.21 | 12% | 88% | | | 0.45 |
| 10.00 | 12% | 88% | | | 0.45 |

The efflux of the HILIC column was led directly to the QqQ-MS for mass spectrometric determination of the hydrophobic compounds of interest (see below)). For the mass spectrometric method used for analyzing the hydrophobic metabolites of interest, the optimized electrospray ionization source parameters were as follows:

| **Parameters** | **Positive mode** | **Negative mode** |
| --- | --- | --- |
| Gas Temperature, °C | 200 | 200 |
| Gas flow, l/min | 13 | 13 |
| Nebuliser, psi | 40 | 40 |
| Sheath Gas Heater | 400 | 400 |
| Sheath Gas Flow | 12 | 12 |
| Capillary, V | 2500 | 3000 |
| V Charging | 300 | 300 |

# The specific LC-MRM parameter sets established for the hydrophilic metabolites and associated SIL-IS together with some instrument specific ionization source settings are presented in S3 Table.
