# Supplementary material for: Prediction of preeclampsia risk in first time pregnant women: Metabolite biomarkers for a clinical test
Source: PLoS One. 2020 Dec 28;15(12):e0244369. doi: 10.1371/journal.pone.0244369 (PMC7769282; doi:10.1371/journal.pone.0244369)
Supplement: S5 File — (DOCX) [file pone.0244369.s005.docx]

# **S5 File. Experimental details of Case-Control Study**

## *Study Preparation*

EDTA plasma samples were retrieved from -80°C storage and thawed on ice in batches of 96, manually transferred to 2D bar-coded 0.65 ml cryovials (Wilmut, Barcelona, Spain) and temporarily organised in a bar-coded 96-position plates (8 x12 positions for individual cryovials; Standard SBS 96-well format, Wilmut), ensuring a transfer of at least 185 µL per sample. Using the Bravo liquid handler, four identical plates with 40 µL sub-aliquots of plasma were generated; per original patient sample up to 40 µL of residual plasma was pooled in a single recipient to create a study QC-pool. The sub-aliquot plates and study QC-pool were then transferred back to -80°C storage. This process was repeated until all study samples were sub-aliquoted. At each cycle, the residual plasmas collected for the study-QC pool were added to the frozen stock of previous rounds. Upon collection of all plasma residuals, the study pool QC was thawed on ice, vortexed, and sub-aliquoted in four sets of 96 x 40 µL study-QC pool aliquots, as per the above protocol, and then returned to -80°C storage till future use.

To inform assay linearity, calibration curves were prepared by fortification of a commercially sourced EDTA plasma with a pre-determined mixture of metabolite reference materials (S4 Table), giving rise to Calibrator Cal1, followed by serial dilution to create 8 Calibrator levels. Calibrators were created in sufficient volumes and multiple sets of 40 µL calibrators were prepared in 0.65 mL cryovials (2D barcoded, Wilmut) and organised in 96 well plates (barcoded, Wilmut), with 12 x 8 calibration sets per plate; and stored at -80°C until future use.

Analytical Quality Controls were created in a similar way: high QC (QCH), and low QC (QCL), respectively corresponding to 80%, and 8% of Cal1, were prepared, sub-aliquoted and stored at -80°C as per above.

*Sample Preparation*

Sample preparation was done in a semi-automated fashion, with all liquid handling steps performed by the Bravo robot, at a rate of one batch / day. Preparation involved the following steps: (1) thawing; (2) fortification of all samples with Internal Standard (IS) mixture containing Stable Isotope Labelled (SIL) metabolites (S5 Table); (3) a protein precipitation-metabolite extraction step using a proprietary formulated extraction solvent in conjunction with low temperature -20°C and centrifugation; (4) distribution of the supernatants (the metabolite extracts) in two duplicate plates, and (5) drying of the extracts by means of vacuum centrifugation. One duplicate plate was returned to the Bravo robot for solvent re-constitution to prepare the plate for RPLC-MS/MS. The other plate was transferred to -80°C until analysis by HILIC-MS/MS.

*Calibrators and QC materials*

Calibrators**:** A calibration curve was established by fortifying a representative sample matrix with the metabolites of interest (“fortification spike mixture”), desirably to a level higher as expected within a specific study population (here pregnant women), followed by a serial dilution of the latter fortified matrix with a relevant diluent, to generate eight different calibration levels. The metabolites levels for the fortified matrix were estimated based on preliminary evaluations carried out (literature review, preliminary studies and preliminary assessments). The concentration span for the calibration curve, defining the dilution series, were estimated also based on preliminary studies and preliminary assessments. Calibrator matrix: Technopath plasma 2% K_2_-EDTA anticoagulant (Technopath, Tipperary, Ireland); diluent: PBS/BSA Buffer (0.01M saline phosphate buffer containing 0.5% bovine Serum Albumin). (PBS and BSA from Sigma Aldrich, Wicklow, Ireland; H_2_O in-house ultrapure water Type1 (@ 18MΩ)). Fortification Spike Mixture: The following fortification spike mixture was prepared by dissolving reference materials for the metabolites of interest in the solvent specified in S5 Table. Dilutions of the stock solutions were made to obtain a fortification spike mix which, when spiked into the calibrator matrix provided the desired concentrations, 300µl of fortification spike mix was thus added to 2700 µL of Technopath plasma.

Calibration curve: Calibrator 1 was prepared by weighing 22.18g of Technopath plasma (approximately 21.6 mL) and adding 2.4 mL of the fortification spike mixture; this solution constituted the highest calibration point (Cal 1) and it was considered to have a 100% concentration (measured as relative concentration). The rest of the calibration curve solutions were prepared by serially dilutions, as exemplified in table below, using the above diluent.

| **Cal level (Cal x)** | **Volume of Cal (x-1)**  **(ml)** | **Volume of diluent (ml)** | **Relative concentration** |
| --- | --- | --- | --- |
| Cal 1 | 24 | 0 | 100% |
| Cal 2 | 4 | 2 | 66.66% |
| Cal 3 | 4 | 2 | 44.44% |
| Cal 4 | 4 | 2 | 29.63% |
| Cal 5 | 4 | 2 | 19.75% |
| Cal 6 | 4 | 2 | 13.17% |
| Cal 7 | 4 | 2 | 8.78% |
| Cal 8 | 4 | 2 | 5.82% |

QC samples:

(1) Study QC Pool samples: Study QC samples were derived from a pooled sample which constituted a pool of specimens contributing to the study. Study QC samples were 40 μL aliquots of this pooled specimen; following their creation they were stored at -80°C till use. Each batch of specimens being processed (from extraction to data analysis) contained 9 aliquots of these Study QC samples.

(2) QC High Samples (QCH): QCH were derived from a dilution of Cal 1 to a level of 80% of Cal 1 using the diluent; again 40 μL aliquots were prepared and stored at -80°C until use. (3) QC Low Samples (QCL): QCL were derived from a dilution of Cal 1 to a level of 8% of Cal 1 using the diluent; again 40 μL aliquots were prepared and stored at -80°C until use.

## *Configuration Analytical Batch*


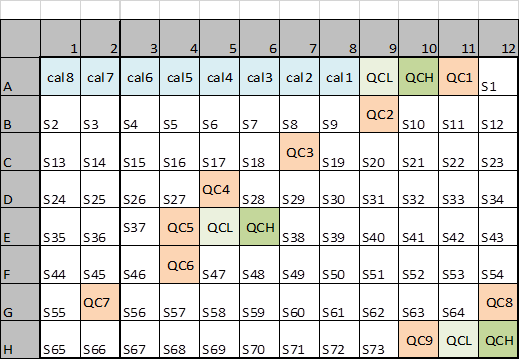
Configuration of a typical analytical batch. SX corresponds a specimen extract (inclusive duplicates), Calx corresponds a calibrator level (8 lowest level – 1 highest level), QC represents a study QC, QCL corresponds to a low level QC sample, and QCH corresponds to a high level QC sample.

## *SIL-IS make up*

The SIL-IS mix was prepared by dissolving the available SIL-IS in the solvent specified in table below and making the necessary dilutions to obtain an SIL-IS mix that when spiked into plasma (10 µL of SIL-IS mix to be spiked onto 40 µL of sample) would provide the desired concentrations in plasma. 40 mL of SIL-IS mixture was prepared; the 40 mL was made up with 50:50 MeOH:H_2_O. Upon preparation, 1200 μL aliquots were created (serving 1 batch of 96 specimens) and stored at -20°C until use. SIL-IS were purchased from: Fluka (Arklow, Ireland), Fischer scientific (Blanchardstown, Ireland), IsoSciences (King of Prussia, PA, USA), Sigma-Aldrich (Wicklow, Ireland), Avanti Lipids (Alabaster, Alabama, USA), QMX Laboratories (Thaxted, UK), LGC (Teddington, U.K), Alfa Chemistry (Holtsville, NY, USA), Generon (Maidenhead, UK), Larodan (Solna, Sweden) and R&D Systems (Abingdon, UK). Depending on physicochemical characteristics of the metabolite of interest, sometimes a salt form of the metabolite of interest was procured.

## *Batch Preparation Protocol*

The following steps were performed for each batch of 96 40μL aliquots; partial batches (n<96) were processed identically:

A 96-position plate (8 x12 positions, PN: W000059X, Wilmut, Barcelona, Spain) with pre-ordered and 40 μL pre-aliquoted specimens (0.65 ml cryovials, PN: W2DST, Wilmut, Barcelona, Spain), constituting an analytical batch, was retrieved from -80 °C storage, and put on BRAVO deck (orbital shaker) and vortexed for 20 minutes to assist thawing. When thawed, the vials were de-capped (manually).

In the meantime: a) A pre-prepared SIL-IS aliquot was retrieved from -20°C storage for thermal conditioning; the SIL-IS was then vortexed (1 minute) and sonicated (5 minutes), and appropriate volumes were pipetted in one column (8 wells) of a PolyPropylene (PP) 96 well plate. The SIL-IS plate was then placed on the BRAVO deck (Peltier at 4°C); b) The pre-prepared proprietary protein precipitation-metabolite extraction formulation (“crash”) stock was taken from -20°C storage, stirred, and a PP 96 well plate filled with the appropriate volumes; the “crash” plate was then put on the robot deck.

The robot protocol was then initiated, the critical steps of this process were:

1. 140 µL of SIL-IS was drawn up from the filled column of the SIL-IS plate and sequentially dispensed: 10µL in each of the specimen vials followed by 15µL of 0.2% formic acid;
2. Fortified specimens were then vortexed, on deck, for 5 min at 1200 rpm;
3. 25 µL of each sample was transferred to another 96-position plate (Wilmut, Barcelona, Spain) containing cyrovials (0.65 mLcryovials, Wilmut, Barcelona, Spain). The original plates, containing 35 µL per / sample, were stored at -80°C for an alternative sample preparation procedure (not used herein);
4. Addition of 200 µL “crash” solution, followed by on deck vortexing for 1 minute at 1200 rpm;
5. The sample plate was then removed from the BRAVO robot and vortexed at 4°C for 10min followed by 2min sonication, and transferred to -20°C for 20 minutes to maximize protein precipitation;
6. After precipitation, the sample vials were centrifuged at 4°C for 20min at a speed of 8000 rpm, then they were returned to the BRAVO robot; the specimen plate was then put on the Peltier station at 4°C;
7. The supernatant was split in 2 sub-aliquots: 240 µL of supernatant was aspirated and 120 µL was dispensed into separate PP 96-well plates, i.e., to enable analyses of the samples by either RPLC-ESI-MS/MS and HILIC-ESI-MS/MS respectively;
8. The specimen extract plates were then dried by means of vacuum evaporation at 40°C for 60 minutes.

One dried extract plate was transferred to -80°C until further analysis, the other extract plate was returned to the BRAVO robot for re-constitution, readying the extracted specimens for LC-MS/MS analysis; Reconstitution was done with 60µL MeOH:ACN:IPA:200mM NH_4_OAc at pH 4.5 (35:35:25:5) or with 60µl H_2_O:MeOH:200mM NH_4_OAc at pH 4.5, (92:3:5) for RPLC- and HILIC- ESI-MS/MS respectively, and then vortexed on deck for 5 minutes, followed by sonication (1 min). For extract plates retrieved from -80°C, a 20 min on deck thermal conditioning step was applied prior to reconstitution.
