# Supplementary material for: Prediction of preeclampsia risk in first time pregnant women: Metabolite biomarkers for a clinical test
Source: PLoS One. 2020 Dec 28;15(12):e0244369. doi: 10.1371/journal.pone.0244369 (PMC7769282; doi:10.1371/journal.pone.0244369)
Supplement: S1 Table — (DOCX) [file pone.0244369.s006.docx]

**S1 Table. Primary metabolite inputs**

| **Original metabolite identification  (Kenny et al. 2010)** | **Representative Metabolites considered in present study** | **CAS-number** | **Reference material available** |
| --- | --- | --- | --- |
| Isobutyrylglycine and/or  N-butyrylglycine | Isobutyrylglycine | 46-98-0 | YES |
|  | N-butyrylglycine | 20208-73-05 | YES |
| Taurine | Taurine | 107-35-7 | YES |
| 5-Hydroxytryptophan | 5-Hydroxytryptophan | 4350-09-8 | YES |
| Urea | Urea | 57-13-6 | YES |
| 12-Ketodeoxycholic acid* | 12-ketodeoxycholic Acid | 5130-29-0 | NO |
| Monosaccharide(s) | D-Glucose | 50-99-7 | YES |
| Sedoheptulose | Sedoheptulose | 3019-74-7 | YES |
| Palmitoylcarnitine | l-Palmitoylcarnitine | 6865-14-1 | YES |
| Stearoylcarnitine | Stearoylcarnitine | 1976-27-8 | YES |
| Decanoylcarnitine | Decanoylcarnitine | 1492-27-9 | YES |
| Octanoylcarnitine | Octanolycarnitine | 25243-95-2 | Not received in time for study |
| Acetylcarnitine | L-Acetylcarnitine | 3040-38-8 | YES |
| Dodecanoylcarnitine | Dodecanoyl-l-carnitine | 25518-54-1 | YES |
| Methylglutaric acid and/or  adipic acid* | 2-methylglutaric acid | 617-62-9 | YES |
|  | 3-methylglutaric acid | 626-51-7 | YES |
|  | Adipic acid | 124-04-9 | YES |
| 8,11,14-Eicosatrienoic acid | 8,11,14 Eicosatrienoic acid | 1783-84-2 | YES |
| 20-Carboxyleukotriene B4 | 20-carboxyleukotriene-B4 | 80434-82-8 | YES |
| Eicosapentaenoic acid and/or  retinoic acid | Eicosapentaenoic acid | 10417-94-4 | YES |
|  | Retinoic acid | 302-79-4 | YES |
| Isovaleric acid and/or  Valeric acid | Isovaleric acid | 503-74-2 | YES |
|  | Valeric acid | 109-52-4 | YES |
| Oleic acid | Oleic acid | 112-80-1 | YES |
| Linoleic acid | Linoleic acid | 60-33-3 | YES |
| Docosahexaenoic acid and/or  docosatriynoic acid | Docosahexaenoic acid | 6217-54-5 | YES |
|  | Docosatriynoic acid | N/A | NO |
| Hydroxy-octadecanoic acid and/or oxo-octadecanoic acid | Hydroxy-octadecenoic acid (Ricinoleic acid) | 141-22-0 | YES |
|  | 13-Oxo-octadecanoic acid | 2389-06-2 | YES |
| Hexadecanoic acid | Hexadecanoic acid (palmitic acid) | 57-10-3 | YES |
| Eicosatetraenoic acid | Arachidonic acid | 506-32-1 | YES |
| Octadecanoic acid | Octadecanoic acid (stearic acid) | 57-11-4 | YES |
| ɣ-Butyrolactone and/or  oxolan-3-one | Oxolan-3-one | 22929-52-8 | YES |
|  | ɣ-Butyrolactone | 46-98-0 | YES |
| 2-Oxovaleric acid and/or oxo-methylbutanoic acid | 2-oxo-3-methylbutanoic acid | 759-05-7 | YES |
|  | 2-Oxovaleric acid | 1821-02-9 | YES |
| 3-hydroxybutanoic acid and/or 2-hydroxybutanoic acid | 2-hydroxybutanoic acid | 5094-24-6 | YES |
|  | 3-hydroxybutanoic acid | 300-85-6 | YES |
| Oxo-tetradecanoic acid and/or hydroxytetradecenoic acid* | 3-hydroxytetradecanoic acid | 3422-31-9 | YES |
|  | 2-hydroxytetradecanoic acid | 2507-55-3 | YES |
|  | 3-Oxotetradecanoic acid | N/A | YES |
| Acetoacetic acid | Acetoacetic acid | 541-50-4 | YES |
| Oxoheptanoic acid | 6-oxoheptanoic acid | 3128-07-2 | YES |
| Di-(heptadecadienoyl)- eicosanoyl-sn-glycerol* | Di-(heptadecadienoyl)-eicosanoyl-sn-glycerol | N/A | NO |
| Hexadecenoyleicosatetraenoyl- sn-glycerol* | Hexadecenoyl-eicosatetraenoyl-sn-glycerol | N/A | NO |
| Di-(octadecadienoyl)-sn-glycerol* | 1,3-Dilinoleoyl-rac-glycerol | 15818-46-9 | YES |
|  | 1,2-Dilinoleoyl-glycerol (1,2-Dilinolein) | 2442-62-8 | YES |
| Octadecenoyl-hexadecanoyl- sn-glycero-3-phosphoserine* | 2-oleoyl-1-palmitoyl-sn-glycero-3-phospho-l-serine | 40290-44-6 | YES |
| Octadecenoyl-sn-glycero- 3-phosphoserine* | Octadecenoyl-sn-glycero-3-phosphoserine | 326589-90-6  (Na salt) | YES |
| Dioctanoyl-sn-glycero-3- phosphocholine* | 1,2-Dioctanoyl-sn-glycero-3-phosphocholine | 19191-91-4 | YES |
| Sphingosine 1-phosphate | Sphingosine 1-phosphate | 26993-306 | YES |
| Sphinganine 1-phosphate | Sphinganine 1-phosphate (C18 base) | 19794-97-9 | NO – inferred† |
|  | Sphinganine 1-phosphate (C17 base) | 474923-29-0 | YES |
| Bilirubin | Bilirubin | 635-65-4 | YES |
| Biliverdin | Biliverdin | 114-25-0 | YES |
| Heme | Heme B | ‎14875-96-8 | NO |
| Vitamin D3 derivatives | 25α-hydroxyvitamin d3 | 63283-36-3 | YES |
| Steroid and/or etiocholan-3-ɑ-o17-one 3-glucuronide* | Etiocholan-3-ɑ-ol-17-one 3 glucuronide | 03/09/3602 | YES |
| **Additional Targets** | **Representative reference and/or rationale** | **CAS-Number** | **Availability** |
| l-Alanine | [1] | 56-41-7 | YES |
| l-Leucine | [1] | 61-90-5 | YES |
| Choline | [1] | 62-49-7 | YES |
| Glycylglycine | [2] | 556-50-3 | YES |
| l-isoleucine | [1] | 73-32-5 | YES |
| l-methionine | [1] | 63-68-3 | YES |
| l-asparagine | [2] | 70-47-3 | YES |
| L-Lysine | [3] | 56-87-1 | YES |
| L-Glutamine | [3] | 56-85-9 | YES |
| l-Arginine | [4] | 74-79-3 | YES |
| Citrulline | [5] | 372-75-8 | YES |
| Homo-L-arginine | [4] | 156-86-5 | YES |
| NG-Monomethyl-L-arginine | [6] | 17035-90-4 | YES |
| Asymmetric dimethylarginine | [4] | 30315-93-6 | YES |
| Symmetric dimethylarginine | [6] | 30344-004 | YES |
| 1-Palmitoyl-2-hydroxy-sn-glycero-  3-phosphocholine (LysoPC(16:0)) | Putative Small for Gestational Age biomarker [7] | 17364-16-8 | YES |
| 6-Hydroxysphingosine§ | Putative Small for Gestational Age biomarker [7] | N/A | YES |
| Cotinine | Biomarker for smoking status [8] | 486-56-6 | YES |
| Myristic acid | [9] | 544-63-8 | YES |
| Ergothioneine | [10] | 497-30-3 | YES |

*Metabolite identification included other similar metabolites of the same class; N/A not available; § Reference material kindly made available by Prof. Overkleeft, Leiden University, The Netherlands; † Kept as an input based on ability to predict assay parameters based on Sphingosine-1-phosphate and literature.

**References**

1. Bahado-Singh RO, Syngelaki A, Akolekar R, Mandal R, Bjondahl TC, Han B, et al. Validation of Metabolomic models for prediction of Early Onset Preeclampsia. Am J Obstet Gynecol. 2015;

2. Kuc S, Koster MPH, Pennings JL a., Hankemeier T, Berger R, Harms AC, et al. Metabolomics Profiling for Identification of Novel Potential Markers in Early Prediction of Preeclampsia. PLoS One. 2014;9(5):e98540.

3. Evans RW, Powers RW, Ness RB, Cropcho LJ, Daftary AR, Harger GF, et al. Maternal and fetal amino acid concentrations and fetal outcomes during pre-eclampsia. Reproduction. 2003;125(6):785–90.

4. Khalil a a, Tsikas D, Akolekar R, Jordan J, Nicolaides KH. Asymmetric dimethylarginine, arginine and homoarginine at 11-13 weeks’ gestation and preeclampsia: a case-control study. J Hum Hypertens. 2013;27(1):38–43.

5. Powers R, Weissgerber TL, McGonigal S, Myerski A, Gallaher M, Speer PD, et al. L-Citrulline administration increases the arginine/ADMA ratio, decreases blood pressure and improves vascular function in obese pregnant women. Pregnancy Hypertens An Int J Women’s Cardiovasc Heal. 2015;5(1):4.

6. Bassareo PP, Mussap M, Bassareo V, Flore G, Mercuro G. Nitrergic system and plasmatic methylarginines: Evidence of their role in the perinatal programming of cardiovascular diseases. Clin Chim Acta. 2015;451(June 2016):21–7.

7. Horgan RP, Broadhurst DI, Walsh SK, Dunn WB, Brown M, Roberts CT, et al. Metabolic profiling uncovers a phenotypic signature of small for gestational age in early pregnancy. J Proteome Res. 2011;10(8):3660–73.

8. Kvalvik LG, Nilsen RM, Skjærven R, Vollset SE, Midttun O, Ueland PM, et al. Self-reported smoking status and plasma cotinine concentrations among pregnant women in the Norwegian Mother and Child Cohort Study. Pediatr Res. 2012;72(1):101–7.

9. Robinson NJ, Minchell LJ, Myers JE, Hubel C a, Crocker IP. A potential role for free fatty acids in the pathogenesis of preeclampsia. J Hypertens. 2009;27(6):1293–302.

10. Turner E, Brewster JA, Simpson NAB, Walker JJ, Fisher J. Imidazole-Based Erythrocyte Markers of Oxidative Stress in Preeclampsia—An NMR Investigation. Reprod Sci. 2009;16(11):1040–51.
