# Supplementary material for: Prediction of preeclampsia risk in first time pregnant women: Metabolite biomarkers for a clinical test
Source: PLoS One. 2020 Dec 28;15(12):e0244369. doi: 10.1371/journal.pone.0244369 (PMC7769282; doi:10.1371/journal.pone.0244369)
Supplement: S2 Table — (DOCX) [file pone.0244369.s007.docx]

**S2 Table. LC-MRM parameters and instrument specific ionization source settings for the hydrophobic metabolites and associated SIL-IS.**

| **Metabolite** | **Rt (min)** | **Quant/ Qual** | **MS1 m/z (Res)** | **MS2 m/z (Res)** | **Quant/ Qual ratio** | **Dwell (ms)** | **Frag (V)** | **CE**  **(V)** | **CAV (V)** | **Polarity** |  |
| --- | --- | --- | --- | --- | --- | --- | --- | --- | --- | --- | --- |
| Decanoylcarnitine | 4.7 | Quant | 316.1 (Unit) | 60.1 (Unit) | 59.9 | 3 | 190 | 24 | 2 | Positive |  |
|  |  | Qual | 316.1 (Unit) | 257.1 (Unit) |  | 3 | 190 | 12 | 2 | Positive |  |
| Biliverdin | 5.1 | Quant | 583.2 (Unit) | 297.1 (Unit) | 200.7 | 3 | 135 | 35 | 5 | Positive |  |
|  |  | Qual | 583.2 (Unit) | 583.1 (Unit) |  | 3 | 135 | 0 | 5 | Positive |  |
| Dodecanoyl-l-carnitine | 5.4 | Quant | 344.1 (Unit) | 85.1 (Unit) | 37.8 | 3 | 140 | 21 | 3 | Positive |  |
|  |  | Qual | 344.1 (Unit) | 85.0 (Unit) |  | 3 | 140 | 51 | 3 | Positive |  |
| 6‑Hydroxysphingosine | 5.6 | Quant | 316.2 (Unit) | 60.1 (Unit) | 12.3 | 3 | 100 | 10 | 7 | Positive |  |
|  |  | Qual | 316.2 (Unit) | 280.1 (Unit) |  | 3 | 100 | 10 | 7 | Positive |  |
| 3‑Hydroxytetradecanoic acid | 5.8 | Quant | 243.1 (Unit) | 59.1 (Unit) | 2.5 | 3 | 120 | 2 | 2 | Negative |  |
|  |  | Qual | 243.1 (Unit) | 41.1 (Unit) |  | 3 | 120 | 45 | 2 | Negative |  |
| 2‑Hydroxytetradecanoic acid | 5.8 | Quant | 243.1 (Unit) | 197.2 (Unit) | 374.6 | 3 | 120 | 15 | 2 | Negative |  |
|  |  | Qual | 243.1 (Unit) | 243.0 (Unit) |  | 3 | 120 | 0 | 2 | Negative |  |
| Sphinganine-1-phosphate (C17 base) | 6 | Quant | 368.1 (Unit) | 270.0 (Unit) | 16.7 | 3 | 100 | 10 | 4 | Positive |  |
|  |  | Qual | 368.1 (Unit) | 252.0 (Unit) |  | 3 | 100 | 25 | 4 | Positive |  |
| Ricinoleic acid | 6.1 | Quant | 297.2 (Unit) | 183.1 (Unit) | 24.7 | 3 | 120 | 15 | 7 | Negative |  |
|  |  | Qual | 297.2 (Unit) | 279.0 (Unit) |  | 3 | 120 | 10 | 7 | Negative |  |
| Sphingosine-1-phosphate | 6.1 | Quant | 380.1 (Unit) | 264.2 (Unit) | 5.9 | 3 | 100 | 11 | 3 | Positive |  |
|  |  | Qual | 380.1 (Unit) | 362.2 (Unit) |  | 3 | 100 | 11 | 3 | Positive |  |
| Sphinganine-1-phosphate | 6.2 | Quant | 382.0 (Unit) | 284.0 (Unit) | 43.3 | 3 | 100 | 8 | 3 | Positive |  |
|  |  | Qual | 382.0 (Unit) | 266.0 (Unit) |  | 3 | 100 | 12 | 3 | Positive |  |
| L-Palmitoylcarnitine | 6.3 | Quant | 400.2 (Unit) | 60.2 (Unit) | 34.8 | 3 | 110 | 26 | 2 | Positive |  |
|  |  | Qual | 400.2 (Unit) | 341.2 (Unit) |  | 3 | 110 | 17 | 2 | Positive |  |
| 13‑Oxooctadecanoic acid | 6.3 | Quant | 299.4 (Unit) | 281.2 (Unit) | 12.5 | 3 | 100 | 5 | 4 | Positive |  |
|  |  | Qual | 299.4 (Unit) | 111.2 (Unit) |  | 3 | 100 | 10 | 4 | Positive |  |
| 1-oleoyl-2-hydroxy-sn-glycero-3-phospho-L-serine | 6.4 | Quant | 524.4 (Unit) | 339.1 (Unit) | 23.6 | 3 | 120 | 20 | 4 | Positive |  |
|  |  | Qual | 524.4 (Unit) | 506.2 (Unit) |  | 3 | 120 | 10 | 4 | Positive |  |
| Bilirubin | 6.5 | Quant | 585.2 (Unit) | 299.1 (Unit) | 0.7 | 3 | 125 | 20 | 5 | Positive |  |
|  |  | Qual | 585.2 (Unit) | 213.1 (Unit) |  | 3 | 125 | 45 | 3 | Positive |  |
| 1-Palmitoyl-2-hydroxy-sn-glycero-3-phosphocholine (LysoPC(16:0)) | 6.5 | Quant | 496.2 (Unit) | 104.1 (Unit) | 34.3 | 3 | 120 | 10 | 2 | Positive |  |
|  |  | Qual | 496.2 (Unit) | 184.0 (Unit) |  | 3 | 120 | 5 | 2 | Positive |  |
| 25-Hydroxyvitamin D_3_ | 6.6 | Quant | 401.3 (Wide) | 383.3 (Wide) | 23.6 | 20 | 104 | 4 | 2 | Positive |  |
|  |  | Qual | 401.3 (Wide) | 365.3 (Wide) |  | 20 | 104 | 4 | 2 | Positive |  |
| Eicosapentaenoic acid | 6.6 | Quant | 301.1 (Unit) | 257.0 (Unit) | 15.1 | 3 | 120 | 5 | 5 | Negative |  |
|  |  | Qual | 301.1 (Unit) | 59.2 (Unit) |  | 3 | 120 | 15 | 5 | Negative |  |
| (1,2-Dioctanoyl-sn-glycero-3-phosphocoline)  1-heptadecanoyl-glycero-3-phosphocholine | 6.6 | Quant | 510.2 (unit) | 184.0 (unit) | 26.5* | 3 | 120 | 25 | 5 | Positive |  |
|  |  | Qual | 510.2 (unit) | 86.1 (unit) |  | 3 | 120 | 45 | 5 | Positive |  |
| Docosahexaenoic acid | 6.7 | Quant | 327.1 (Unit) | 283.1 (Unit) | 11.8 | 3 | 80 | 5 | 2 | Negative |  |
|  |  | Qual | 327.1 (Unit) | 229.1 (Unit) |  | 3 | 80 | 5 | 2 | Negative |  |
| Myristic acid | 6.7 | Quant | 227.2 (Unit) | 227.1 (Unit) | 0.5 | 3 | 145 | 0 | 4 | Negative |  |
|  |  | Qual | 227.2 (Unit) | 53.8 (Unit) |  | 3 | 145 | 45 | 4 | Negative |  |
| Stearoylcarnitine | 6.7 | Quant | 428.3 (Unit) | 85.0 (Unit) | 2.7 | 3 | 130 | 25 | 5 | Positive |  |
|  |  | Qual | 428.3 (Unit) | 369.3 (Unit) |  | 3 | 130 | 15 | 5 | Positive |  |
| Arachidonic acid | 6.8 | Quant | 303.1 (Unit) | 259.1 (Unit) | 34.7 | 3 | 135 | 3 | 2 | Negative |  |
|  |  | Qual | 303.1 (Unit) | 59.1 (Unit) |  | 3 | 135 | 15 | 2 | Negative |  |
| Linoleic acid | 6.9 | Quant | 279.1 (Unit) | 279.1 (Unit) | 13.8 | 3 | 104 | 10 | 2 | Negative |  |
|  |  | Qual | 279.1 (Unit) | 279.0 (Unit) |  | 3 | 104 | 20 | 2 | Negative |  |
| 8,11,14 Eicosatrienoic acid | 7 | Quant | 305.1 (Unit) | 305.0 (Unit) | 152.7 | 3 | 80 | 1 | 2 | Negative |  |
|  |  | Qual | 305.1 (Unit) | 304.9 (Unit) |  | 3 | 80 | 0 | 2 | Negative |  |
| Hexadecanoic acid | 7 | Quant | 255.1 (Unit) | 255.1 (Unit) | 21.5 | 3 | 130 | 15 | 3 | Negative |  |
|  |  | Qual | 255.1 (Unit) | 255.0 (Unit) |  | 3 | 130 | 20 | 3 | Negative |  |
| Oleic acid | 7.1 | Quant | 281.1 (Unit) | 281.1 (Unit) | 22.3 | 3 | 128 | 10 | 3 | Negative |  |
|  |  | Qual | 281.1 (Unit) | 281.0 (Unit) |  | 3 | 128 | 20 | 3 | Negative |  |
| Etiocholanolone glucuronide | 7.1 | Quant | 465.2 (Unit) | 465.1 (Unit) | Na | 3 | 135 | 0 | 6 | Negative |  |
|  |  | Qual | 465.2 (Unit) | 113.0 (Unit) |  | 3 | 135 | 35 | 6 | Negative |  |
| Stearic acid | 7.4 | Quant | 283.2 (Unit) | 265.0 (Unit) | 6.8 | 3 | 145 | 19 | 2 | Negative |  |
|  |  | Qual | 283.2 (Unit) | 45.1 (Unit) |  | 3 | 145 | 20 | 2 | Negative |  |
| 20-Carboxy-leukotriene B4 | 7.4 | Quant | 365.0 (Unit) | 364.9 (Unit) | 0.5 | 3 | 120 | 0 | 5 | Negative |  |
|  |  | Qual | 365.0 (Unit) | 195.0 (Unit) |  | 3 | 120 | 15 | 5 | Negative |  |
| Dilinoleoyl-glycerol^+^ | 8.8 | Quant | 634.4 (Unit) | 337.5 (Unit) | 185.4 | 3 | 84 | 28 | 2 | Positive |  |
|  |  | Qual | 634.4 (Unit) | 599.2 (Unit) |  | 3 | 84 | 16 | 2 | Positive |  |
| **SIL-IS** | | | | | | | | | | | |
| Decanoylcarnitine-[^2^H_3_] | 4.7 | Quant | 319.1 (Unit) | 63.1 (Unit) | 46.6 | 3 | 190 | 24 | 2 | Positive |  |
|  |  | Qual | 319.1 (Unit) | 257.1 (Unit) |  | 3 | 190 | 12 | 2 | Positive |  |
| Biliverdin [^2^H_4_] | 5.1 | Quant | 586.0 (Unit) | 299.2 (Unit) | NA | 3 | 130 | 35 | 5 | Positive |  |
| Dodecanoyl-l-carnitine-[^2^H_3_] | 5.4 | Quant | 347.1 (Unit) | 85.1 (Unit) | 29.8 | 3 | 140 | 21 | 3 | Positive |  |
|  |  | Qual | 347.1 (Unit) | 85.0 (Unit) |  | 3 | 140 | 51 | 3 | Positive |  |
| Sphingosine-1-phosphate-[^13^C_2_,^2^H_2_] | 6.1 | Quant | 384.2 (Unit) | 268.2 (Unit) | 4.3 | 3 | 100 | 11 | 3 | Positive |  |
|  |  | Qual | 384.2 (Unit) | 366.2 (Unit) |  | 3 | 100 | 11 | 3 | Positive |  |
| Palmitoyl carnitine-[^2^H_3_] | 6.3 | Quant | 403.2 (Unit) | 63.2 (Unit) | 29.7 | 3 | 190 | 5 | 2 | Positive |  |
|  |  | Qual | 403.2 (Unit) | 341.2 (Unit) |  | 3 | 190 | 0 | 2 | Positive |  |
| Bilirubin [^2^H_4_] | 6.5 | Quant | 590.2 (Unit) | 301.2 (Unit) | 98.8 | 3 | 125 | 15 | 5 | Positive |  |
|  |  | Qual | 590.2 (Unit) | 303.2 (Unit) |  | 3 | 125 | 15 | 5 | Positive |  |
| 25-Hydroxyvitamin D_3_-[^2^H_3_] | 6.6 | Quant | 404.2 (Wide) | 386.2 (Wide) | 46.6 | 20 | 98 | 10 | 2 | Positive |  |
|  |  | Qual | 404.2 (Wide) | 368.3 (Wide) |  | 20 | 98 | 10 | 2 | Positive |  |
| Stearoyl-L-carnitine [^2^H_3_] | 6.7 | Quant | 431.3 (Unit) | 85.0 (Unit) | 2.7 | 3 | 130 | 25 | 5 | Positive |  |
|  |  | Qual | 431.3 (Unit) | 369.3 (Unit) |  | 3 | 130 | 15 | 5 | Positive |  |
| Docosahexaenoic acid-[^2^H_5_] | 6.7 | Quant | 332.1 (Unit) | 288.1 (Unit) | 12.1 | 3 | 80 | 5 | 2 | Negative |  |
|  |  | Qual | 332.1 (Unit) | 234.1 (Unit) |  | 3 | 80 | 5 | 2 | Negative |  |
| Arachidonic acid-[^2^H_8_] | 6.8 | Quant | 311.1 (Unit) | 267.1 (Unit) | 36.1 | 3 | 135 | 3 | 2 | Negative |  |
|  |  | Qual | 311.1 (Unit) | 59.1 (Unit) |  | 3 | 135 | 15 | 3 | Negative |  |
| Linoleic acid-[^13^C_18_] | 6.9 | Quant | 297.3 (Unit) | 297.3 (Unit) | 21.5 | 3 | 104 | 10 | 3 | Negative |  |
|  |  | Qual | 297.3 (Unit) | 297.2 (Unit) |  | 3 | 104 | 20 | 3 | Negative |  |
| Hexadecanoic acid-[^2^H_4_] | 7 | Quant | 259.1 (Unit) | 259.1 (Unit) | 24.1 | 3 | 130 | 15 | 3 | Negative |  |
|  |  | Qual | 259.1 (Unit) | 259.0 (Unit) |  | 3 | 130 | 20 | 3 | Negative |  |
| Oleic acid-[^13^C_5_] | 7.1 | Quant | 286.3 (Unit) | 286.3 (Unit) | 24.5 | 3 | 128 | 10 | 3 | Negative |  |
|  |  | Qual | 286.3 (Unit) | 286.2 (Unit) |  | 3 | 128 | 20 | 3 | Negative |  |
| 1,3-Dilinoleoyl-rac-glycerol-[^2^H_5_] | 8.8 | Quant | 639.4 (Unit) | 342.5 (Unit) | 141.1 | 3 | 84 | 20 | 2 | Positive |  |
|  |  | Qual | 639.4 (Unit) | 604.2 (Unit) |  | 3 | 84 | 10 | 2 | Positive |  |

^+^ Read-out is a combined signal of 1,3-rac-Dilinoleoyl-glycerol and 1,2-rac-Dilinoleoyl-glycerol; * quant / qual ratio derived from 1-heptadecanoyl-glycero-3-phosphocholine in samples; value confirmed post study using reference material
