# Supplementary material for: Prediction of preeclampsia risk in first time pregnant women: Metabolite biomarkers for a clinical test
Source: PLoS One. 2020 Dec 28;15(12):e0244369. doi: 10.1371/journal.pone.0244369 (PMC7769282; doi:10.1371/journal.pone.0244369)
Supplement: S3 Table — (DOCX) [file pone.0244369.s008.docx]

**S3 Table. LC-MRM parameters and instrument specific ionization source settings for the hydrophilic metabolites and associated SIL-IS.**

| **Metabolite** | **Rt (min)** | **Quant/ Qual** | **MS1 m/z (Res)** | **MS2 m/z (Res)** | **Quant/ Qual ratio** | **Dwell (ms)** | **Frag (V)** | **CE (V)** | **CAV (V)** | **Polarity** |
| --- | --- | --- | --- | --- | --- | --- | --- | --- | --- | --- |
| Urea | 1.3 | Quant | 61.2 (Unit) | 44.3 (Unit) | 167.3 | 3 | 100 | 10 | 2 | Positive |
|  |  | Qual | 61.2 (Unit) | 61.2 (Unit) |  | 3 | 100 | 10 | 2 | Positive |
| Cotinine | 1.3 | Quant | 177.0 (Unit) | 80.0 (Unit) | 19.7 | 3 | 100 | 25 | 5 | Positive |
|  |  | Qual | 177.0 (Unit) | 98.1 (Unit) |  | 3 | 100 | 20 | 5 | Positive |
| 2-Hydroxybutanoic acid | 2.3 | Quant | 103.0 (Unit) | 57.2 (Unit) | 14 | 15 | 84 | 8 | 4 | Negative |
|  |  | Qual | 103.0 (Unit) | 45.2 (Unit) |  | 15 | 84 | 5 | 4 | Negative |
| 3-Hydroxybutanoic acid | 2.7 | Quant | 103.1 (Unit) | 59.1 (Unit) | 344.9 | 15 | 78 | 8 | 4 | Negative |
|  |  | Qual | 103.1 (Unit) | 103.1 (Unit) |  | 15 | 78 | 0 | 4 | Negative |
| Taurine | 2.9 | Quant | 126.1 (Unit) | 44.2 (Unit) | 35.7 | 3 | 100 | 20 | 2 | Positive |
|  |  | Qual | 126.1 (Unit) | 108.0 (Unit) |  | 3 | 100 | 10 | 2 | Positive |
| Isobutyrylglycine | 3 | Quant | 146.0 (Unit) | 76.2 (Unit) | 136.7 | 3 | 60 | 5 | 7 | Positive |
|  |  | Qual | 146.0 (Unit) | 43.2 (Unit) |  | 3 | 60 | 15 | 7 | Positive |
| L-Leucine | 3.4 | Quant | 132.0 (Unit) | 86.2 (Unit) | 19.6 | 3 | 104 | 10 | 4 | Positive |
|  |  | Qual | 132.0 (Unit) | 44.2 (Unit) |  | 3 | 104 | 25 | 4 | Positive |
| L-Methionine | 3.5 | Quant | 150.0 (Unit) | 56.2 (Unit) | 37 | 3 | 104 | 16 | 2 | Positive |
|  |  | Qual | 150.0 (Unit) | 104.1 (Unit) |  | 3 | 104 | 14 | 2 | Positive |
| L-Isoleucine | 3.6 | Quant | 132.0 (Unit) | 69.2 (Unit) | 22.9 | 3 | 104 | 19 | 2 | Positive |
|  |  | Qual | 132.0 (Unit) | 57.2 (Unit) |  | 3 | 104 | 32 | 2 | Positive |
| L-Alanine | 4.6 | Quant | 90.1 (Unit) | 90.1 (Unit) | 74.7 | 3 | 62 | 0 | 2 | Positive |
|  |  | Qual | 90.1 (Unit) | 44.1 1 (Unit) |  | 3 | 62 | 8 | 2 | Positive |
| 2-Methylglutaric acid | 4.8 | Quant | 145.0 (Unit) | 101.2 (Unit) | 9.4 | 15 | 80 | 8 | 4 | Negative |
|  |  | Qual | 145.0 (Unit) | 83.2 (Unit) |  | 15 | 80 | 12 | 4 | Negative |
| L-(+)-Ergothioneine | 4.8 | Quant | 230.1 (Unit) | 127.0 (Unit) | 47.3 | 3 | 100 | 25 | 2 | Positive |
|  |  | Qual | 230.1 (Unit) | 186.0 (Unit) |  | 3 | 100 | 15 | 2 | Positive |
| L-Glutamine | 4.8 | Quant | 144.9 (Unit) | 127.0 (Unit) | 31.8 | 3 | 100 | 10 | 2 | Negative |
|  |  | Qual | 144.9 (Unit) | 108.8 (Unit) |  | 3 | 100 | 15 | 2 | Negative |
| Citrulline | 5 | Quant | 176.0 (Unit) | 113.0 (Unit) | 261.2 | 3 | 68 | 15 | 5 | Positive |
|  |  | Qual | 176.0 (Unit) | 70.1 (Unit) |  | 3 | 68 | 20 | 5 | Positive |
| Adipic acid | 5.1 | Quant | 145.1 (Unit) | 83.2 (Unit) | 221.7 | 3 | 80 | 12 | 4 | Negative |
|  |  | Qual | 145.1 (Unit) | 101.2 (Unit) |  | 3 | 80 | 8 | 4 | Negative |
| Glycyl-glycine | 5.4 | Quant | 133.1 (Unit) | 30.4 (Unit) | 15.7 | 3 | 58 | 20 | 4 | Positive |
|  |  | Qual | 133.1 (Unit) | 76.2 (Unit) |  | 3 | 58 | 5 | 4 | Positive |
| Choline | 5.7 | Quant | 104.1 (Unit) | 45.3 (Unit) | 171.5 | 3 | 40 | 27 | 2 | Positive |
|  |  | Qual | 104.1 (Unit) | 60.3 (Unit) |  | 3 | 40 | 17 | 2 | Positive |
| L-Acetylcarnitine | 6.1 | Quant | 204.2 (Unit) | 60.1 (Unit) | 377.5 | 3 | 100 | 15 | 4 | Positive |
|  |  | Qual | 204.2 (Unit) | 85.0 (Unit) |  | 3 | 100 | 15 | 4 | Positive |
| L-Arginine | 6.8 | Quant | 175.0 (Unit) | 116.0 (Unit) | 505.9 | 3 | 82 | 15 | 2 | Positive |
|  |  | Qual | 175.0 (Unit) | 70.1 (Unit) |  | 3 | 82 | 20 | 2 | Positive |
| Homo-L-arginine | 6.8 | Quant | 189.0 (Unit) | 144.2 (Unit) | 2.6 | 3 | 88 | 15 | 2 | Positive |
|  |  | Qual | 189.0 (Unit) | 57.1 (Unit) |  | 3 | 88 | 25 | 2 | Positive |
| NG-Monomethyl-L-arginine | 7.2 | Quant | 189.0 (Unit) | 116.2 (Unit) | 180.2 | 3 | 88 | 15 | 2 | Positive |
|  |  | Qual | 189.0 (Unit) | 70.2 (Unit) |  | 3 | 88 | 15 | 2 | Positive |
| L-Lysine | 7.4 | Quant | 146.9 (Unit) | 130.2 (Unit) | 613.4 | 1 | 100 | 20 | 2 | Positive |
|  |  | Qual | 146.9 (Unit) | 84.2 (Unit) |  | 1 | 100 | 2 | 2 | Positive |
| Symmetric dimethylarginine | 7.7 | Quant | 203.1 (Unit) | 172.2 (Unit) | 49.3 | 3 | 90 | 10 | 4 | Positive |
|  |  | Qual | 203.1 (Unit) | 133.0 (Unit) |  | 3 | 90 | 6 | 4 | Positive |
| Asymmetric dimethylarginine | 7.9 | Quant | 203.0 (Unit) | 46.2 (Unit) | 188.5 | 3 | 100 | 15 | 4 | Positive |
|  |  | Qual | 203.0 (Unit) | 70.1 (Unit) |  | 3 | 100 | 18 | 4 | Positive |
| **SIL-IS** | | | | | | | | | | |
| Urea [^13^C, ^18^O] | 1.3 | Quant | 64.2 (Unit) | 47.2 (Unit) | 469.3 | 3 | 100 | 25 | 2 | Positive |
|  |  | Qual | 64.2 (Unit) | 64.1 (Unit) |  | 3 | 100 | 0 | 2 | Positive |
| (±)-Cotinine [^2^H_3_] | 1.4 | Quant | 180.0 (Unit) | 80.0 (Unit) | 21.7 | 3 | 100 | 25 | 5 | Positive |
|  |  | Qual | 180.0 (Unit) | 101.0 (Unit) |  | 3 | 100 | 20 | 5 | Positive |
| 2-Hydroxybutanoic acid -[^2^H_3_] | 2.5 | Quant | 106.0 (Unit) | 59.2 (Unit) | 12.9 | 15 | 84 | 8 | 4 | Negative |
|  |  | Qual | 106.0 (Unit) | 45.2 (Unit) |  | 15 | 84 | 5 | 4 | Negative |
| 3-Hydroxybutanoic acid [^2^H_4_] | 2.7 | Quant | 107.0 (Unit) | 107.0 (Unit) | 26.7 | 15 | 78 | 0 | 3 | Negative |
|  |  | Qual | 107.0 (Unit) | 59.1 (Unit) |  | 15 | 78 | 8 | 3 | Negative |
| Taurine [^13^C_2_] | 2.9 | Quant | 128.1 (Unit) | 46.2 (Unit) | 74.2 | 3 | 102 | 16 | 3 | Positive |
|  |  | Qual | 128.1 (Unit) | 110.2 (Unit) |  | 3 | 102 | 8 | 3 | Positive |
| N-Isobutyrylglycine [^13^C_2_, ^15^N] | 3.1 | Quant | 149.0 (Unit) | 43.2 (Unit) | 82.9 | 3 | 60 | 15 | 7 | Positive |
|  |  | Qual | 149.0 (Unit) | 79.1 (Unit) |  | 3 | 60 | 5 | 7 | Positive |
| Leucine-[^13^C_6_] | 3.4 | Quant | 138.0 (Unit) | 46.2 (Unit) | 10.7 | 3 | 104 | 25 | 4 | Positive |
|  |  | Qual | 138.0 (Unit) | 44.2 (Unit) |  | 3 | 104 | 25 | 4 | Positive |
| L-Methionine-[^13^C_5_] | 3.5 | Quant | 155.0 (Unit) | 59.2 (Unit) | 40 | 3 | 104 | 16 | 2 | Positive |
|  |  | Qual | 155.0 (Unit) | 108.2 (Unit) |  | 3 | 104 | 14 | 2 | Positive |
| Isoleucine-[^13^C_6_] | 3.6 | Quant | 138.0 (Unit) | 74.2 (Unit) | 24.6 | 3 | 104 | 19 | 3 | Positive |
|  |  | Qual | 138.0 (Unit) | 60.2 (Unit) |  | 3 | 104 | 32 | 3 | Positive |
| L-Alanine-[^13^C_3_] | 4.6 | Quant | 93.1 (Unit) | 93.1 (Unit) | 94.5 | 3 | 62 | 0 | 2 | Positive |
|  |  | Qual | 93.1 (Unit) | 46.1 (Unit) |  | 3 | 62 | 8 | 2 | Positive |
| L-Glutamine [^13^C_5_] | 4.7 | Quant | 149.9 (Unit) | 131.9 (Unit) | 24.8 | 3 | 100 | 10 | 2 | Negative |
|  |  | Qual | 149.9 (Unit) | 113.8 (Unit) |  | 3 | 100 | 15 | 2 | Negative |
| 2-Methylglutaric acid [^13^C_2_] | 4.8 | Quant | 147.0 (Unit) | 102.0 (Unit) | 24.8 | 15 | 80 | 10 | 4 | Negative |
|  |  | Qual | 147.0 (Unit) | 84.0 (Unit) |  | 15 | 80 | 10 | 4 | Negative |
| Adipic acid [^2^H_4_] | 4.8 | Quant | 149.0 (Unit) | 105.2 (Unit) | 0.25 | 3 | 80 | 10 | 4 | Negative |
|  |  | Qual | 149.0 (Unit) | 87.2 (Unit) |  | 3 | 80 | 10 | 4 | Negative |
| L-(+)-Ergothioneine [^2^H_9_] | 4.8 | Quant | 239.0 (Unit) | 127.0 (Unit) | 122.3 | 3 | 100 | 25 | 4 | Positive |
|  |  | Qual | 239.0 (Unit) | 195.0 (Unit) |  | 3 | 100 | 10 | 2 | Positive |
| L-Citrulline [^2^H_7_] | 5.1 | Quant | 183.1 (Unit) | 120.1 (Unit) | 231.6 | 3 | 68 | 16 | 5 | Positive |
|  |  | Qual | 183.1 (Unit) | 166.1 (Unit) |  | 3 | 68 | 4 | 5 | Positive |
| Glycyl-glycine [^13^C_4_, ^15^N_2_] | 5.4 | Quant | 138.9 (Unit) | 79.1 (Unit) | 24.5 | 3 | 58 | 5 | 4 | Positive |
|  |  | Qual | 138.9 (Unit) | 32.2 (Unit) |  | 3 | 58 | 20 | 4 | Positive |
| Choline-[^2^H_9_] | 5.7 | Quant | 114.0 (Unit) | 45.2 (Unit) | 141.3 | 3 | 40 | 20 | 2 | Positive |
|  |  | Qual | 114.0 (Unit) | 69.2 (Unit) |  | 3 | 40 | 20 | 2 | Positive |
| L-Acetylcarnitine [^2^H_3_] | 6.1 | Quant | 207.2 (Unit) | 60.1 (Unit) | 853.4 | 3 | 100 | 15 | 4 | Positive |
|  |  | Qual | 207.2 (Unit) | 85.0 (Unit) |  | 3 | 100 | 15 | 4 | Positive |
| L-Arginine-[^13^C_6_] | 6.8 | Quant | 181.2 (Unit) | 61.3 (Unit) | 55.6 | 3 | 82 | 12 | 2 | Positive |
|  |  | Qual | 181.2 (Unit) | 121.1 (Unit) |  | 3 | 82 | 12 | 2 | Positive |
| Homo-L-arginine [^13^C_7_, ^15^N_4_] | 6.8 | Quant | 200.0 (Unit) | 153.0 (Unit) | 717.1 | 3 | 88 | 5 | 2 | Positive |
|  |  | Qual | 200.0 (Unit) | 90.2 (Unit) |  | 3 | 88 | 20 | 2 | Positive |
| Symmetric Dimethylarginine- [^2^H_6_] | 7.7 | Quant | 209.1 (Unit) | 175.1 (Unit) | 41.9 | 3 | 90 | 10 | 4 | Positive |
|  |  | Qual | 209.1 (Unit) | 164.0 (Unit) |  | 3 | 90 | 15 | 4 | Positive |
| Asymmetric dimethylarginine [^2^H_6_] | 7.9 | Quant | 209.2 (Unit) | 52.3 (Unit) | 175 | 3 | 100 | 15 | 4 | Positive |
|  |  | Qual | 209.2 (Unit) | 70.2 (Unit) |  | 3 | 100 | 20 | 4 | Positive |
