# Supplementary material for: Prediction of preeclampsia risk in first time pregnant women: Metabolite biomarkers for a clinical test
Source: PLoS One. 2020 Dec 28;15(12):e0244369. doi: 10.1371/journal.pone.0244369 (PMC7769282; doi:10.1371/journal.pone.0244369)
Supplement: S4 Table — (DOCX) [file pone.0244369.s009.docx]

**S4 Table. Composition and concentrations of fortification spike mixture**

| **Metabolite** | **Individual stock solution**  **(ng/mL)** | **Solvent used** | **HQC Standard mix**  **(µg in 5mL solution)** | **Final concentration Metabolite fortification in matrix**  **(ng/mL)** |
| --- | --- | --- | --- | --- |
| Isobutyrylglycine | 1000000 | MeOH | 4000 | 80 |
| Taurine | 5000000 | H_2_O | 500000 | 10000 |
| Urea | 10000000 | H_2_O | 2200000 | 44000 |
| L-Palmitoylcarnitine | 1000000 | MeOH | 2400 | 48 |
| Stearoylcarnitine | 1000000 | MeOH | 2000 | 40 |
| Decanoylcarnitine | 10000 | H_2_O | 1200 | 24 |
| L-Acetylcarnitine | 1000000 | MeOH | 100000 | 2000 |
| Dodecanoyl-l-carnitine | 10000 | H_2_O | 400 | 8 |
| 2-Methylglutaric acid | 5000000 | MeOH | 15000 | 300 |
| Adipic acid | 5000000 | MeOH | 15000 | 300 |
| 8,11,14 Eicosatrienoic acid | 50000000 | MeOH | 800000 | 16000 |
| 20-Carboxy-leukotriene B4 | 1000000 | EtOH | 4000 | 80 |
| Eicosapentaenoic acid | 10000000 | MeOH | 160000 | 3200 |
| Oleic acid | 5000000 | EtOH | 1150000 | 23000 |
| Linoleic acid | 5000000 | EtOH | 840000 | 16800 |
| Docosahexaenoic acid | 5000000 | EtOH | 200000 | 4000 |
| Ricinoleic acid | 100000000 | MeOH | 20000 | 400 |
| 13‑Oxooctadecanoic acid | 2000000 | MeOH | 12000 | 240 |
| Hexadecanoic acid | 5000000 | EtOH | 800000 | 16000 |
| Arachidonic acid | 5000000 | EtOH | 120000 | 2400 |
| Stearic acid | 6200000 | MeOH | 620000 | 12400 |
| 2-Hydroxybutanoic acid | 5000000 | H_2_O | 120000 | 2400 |
| 3-Hydroxybutanoic acid | 500000 | H_2_O | 27000 | 540 |
| 3‑Hydroxytetradecanoic acid | 1000000 | MeOH | 5000 | 100 |
| 2‑Hydroxytetradecanoic acid | 1000000 | MeOH | 4000 | 80 |
| 1,2-Dilinoleoyl-rac-glycerol | 12000000 | MeOH | 254400 | 5088 |
| 1-oleoyl-2-hydroxy-sn-glycero-3-phospho-L-serine | 5000000 | CHCl_3_ | 112000 | 2240 |
| 1,2-Dioctanoyl-sn-glycero-3-phosphocholine | 1000000 | MeOH | 12000 | 240 |
| Sphingosine-1-phosphate | 1000000 | MeOH | 90000 | 1800 |
| Sphinganine-1-phosphate (C17 base) | 1000000 | H_2_O | 20000 | 400 |
| Bilirubin | 1000000 | MeOH | 160000 | 3200 |
| Biliverdin | 1000000 | MeOH | 200000 | 4000 |
| 25-Hydroxyvitamin D3 | 1000000 | EtOH | 12000 | 240 |
| Etiocholanolone glucuronide | 1000000 | MeOH | 20000 | 400 |
| L-Alanine | 5000000 | H_2_O | 800000 | 16000 |
| L-Leucine | 5000000 | H_2_O | 300000 | 6000 |
| Choline | 5000000 | H_2_O | 40000 | 800 |
| Glycyl-glycine | 5000000 | H_2_O | 50000 | 1000 |
| L-Isoleucine | 5000000 | H_2_O | 400000 | 8000 |
| L-Methionine | 5000000 | H_2_O | 300000 | 6000 |
| L-Lysine | 22000000 | H_2_O | 2970000 | 59400 |
| L-Glutamine | 19700000 | H_2_O | 945600 | 18912 |
| L-Arginine | 5000000 | H_2_O | 400000 | 8000 |
| Citrulline | 5000000 | H_2_O | 120000 | 2400 |
| Homo-L-arginine | 5000000 | H_2_O | 80000 | 1600 |
| NG-Monomethyl-L-arginine | 5000000 | H_2_O | 32000 | 640 |
| Asymmetric dimethylarginine | 1000000 | H_2_O | 20000 | 400 |
| Symmetric dimethylarginine | 1000000 | H_2_O | 62500 | 1250 |
| 1-Palmitoyl-2-hydroxy-sn-glycero-3-phosphocholine (LysoPC(16:0)) | 1500000 | MeOH | 20100 | 402 |
| 6‑Hydroxysphingosine | 31549.8 | MeOH | 1262 | 25 |
| Cotinine | 10000000 | MeOH/H_2_O (50/50) | 70000 | 1400 |
| Myristic acid | 10000000 | MeOH | 100000 | 2000 |
| L-(+)-Ergothioneine | 600000 | MeOH | 38400 | 768 |
