# Supplementary material for: Prediction of preeclampsia risk in first time pregnant women: Metabolite biomarkers for a clinical test
Source: PLoS One. 2020 Dec 28;15(12):e0244369. doi: 10.1371/journal.pone.0244369 (PMC7769282; doi:10.1371/journal.pone.0244369)
Supplement: S5 Table — (DOCX) [file pone.0244369.s010.docx]

**S5 Table.** **Composition and concentrations of SIL-IS mixture**

| **SIL-IS** | **CAS** | **Individual stock solution (ng/mL)** | **Solvent used** | **SIL IS**  **(µg in 40 mL solution)** | **SIL-IS concentration in plasma**  **(ng/mL)** |
| --- | --- | --- | --- | --- | --- |
| N-Isobutyrylglycine-[^13^C_2_,^15^N] | n/a | 1000 | H_2_O | 150 | 750 |
| Taurine [^13^C_2_] | CAS 70155-54-3 | 1000 | H_2_O | 500 | 2500 |
| Urea [^13^C, ^18^O] | n/a | 30000 | H_2_O | 3600 | 18000 |
| Palmitoyl carnitine‑[^2^H_3_] | n/a | 1000 | MeOH | 150 | 750 |
| Stearoyl-L-carnitine [^2^H_3_] | CAS 25597-09-5 | 100 | H_2_O | 0.2 | 1 |
| Decanoylcarnitine‑[^2^H_3_] | n/a | 1000 | H_2_O | 0.5 | 2.5 |
| L-Acetylcarnitine [^2^H_3_] | CAS 362049-62-5 | 1000 | H_2_O | 150 | 750 |
| Dodecanoyl‑L‑carnitine‑[^2^H_3_] | CAS 1021439-26-8 | 1000 | H_2_O | 1 | 5 |
| 2‑Methylglutaric‑[^13^C_2_] | CAS 1219798-68-1 | 1000 | MeOH | 200 | 800 |
| Adipic acid-[^2^H_4_] | CAS 19031-55-1 | 1000 | MeOH | 10 | 50 |
| Oleic acid‑[^13^C_5_] | CAS 1255644-48-4 | 10000 | EtOH | 2000 | 10000 |
| Linoleic acid‑[^13^C_18_] | n/a | 10000 | EtOH | 1000 | 5000 |
| Docosahexaenoic acid‑[^2^H_5_] | CAS 1197205-71-2 | 1000 | MeOH | 375 | 1875 |
| Hexadecanoic acid [^2^H_4_] | CAS 75736-49-1 | 10000 | EtOH | 750 | 3750 |
| Arachidonic acid‑[^2^H_8_] | CAS 69254-37-1 | 5000 | EtOH | 500 | 2500 |
| 2-Hydroxybutanoic acid‑[^2^H_3_] | CAS 1219798-97-6 | 1000 | H_2_O | 225 | 1125 |
| 3-Hydroxybutanoic acid-[^2^H_4_] | CAS 1219804-68-8 | 1000 | H_2_O | 200 | 1000 |
| 1,3-Dilinoleoyl-rac-glycerol [^2^H_5_] | CAS 15818-46-9 | 1000 | EtOH | 600 | 3000 |
| Sphingosine 1-phosphate‑[^13^C_2_,^2^H_2_] | n/a | 1000 | MeOH | 200 | 1000 |
| Bilirubin-[^2^H_4_] | n/a | 2500 | MeOH + 0.01% NH_3_ | 2500 | 12500 |
| Biliverdin-[^2^H_4_] | n/a | 1000 | MeOH | 225 | 1125 |
| 25-Hydroxyvitamin D_3_‑[^2^H_3_] | CAS 140710-94-7 | 1000 | EtOH (ethanol) | 90 | 450 |
| L‑Alanine‑[^13^C_3_] | CAS 100108-77-8 | 10000 | H_2_O | 2000 | 10000 |
| Leucine‑[^13^C_6_] | n/a | 1000 | H_2_O | 225 | 1125 |
| Choline [^2^H_9_] | CAS 61037-86-3 | 2000 | H_2_O | 1500 | 7500 |
| Glycyl-glycine [^13^C_4_, ^15^N_2_] | n/a | 1000 | MeOH | 200 | 1000 |
| Isoleucine‑[^13^C_6_] | n/a | 1000 | H_2_O | 750 | 3750 |
| L-methionine‑[^13^C_5_] | n/a | 1000 | H_2_O | 300 | 1500 |
| L-Glutamine [^13^C_5_] | n/a | 12200 | H_2_O | 4880 | 24400 |
| L-Arginine‑[^13^C_6_] | n/a | 1000 | H_2_O | 650 | 3250 |
| L-Citrulline-[^2^H_7_] | n/a | 1000 | H_2_O | 250 | 1250 |
| Homo-L-arginine [^13^C_7_, ^15^N_4_] | n/a | 1000 | H_2_O | 750 | 3750 |
| Asymmetric dimethyl arginine [^2^H_6_] | CAS 1313730-20-9 | 2500 | MeOH | 12.5 | 62.5 |
| Symmetric dimethylarginine‑[^2^H_6_] | 1331888-08-4 | 1000 | H_2_O | 10 | 50 |
| (±)-Cotinine [^2^H_3_] | CAS 110952-70-0 | 1000 | H_2_O | 45 | 225 |
| L-(+)-Ergothioneine [^2^H_9_] | n/a | 1000 | H_2_O | 1000 | 5000 |
