# Supplementary material for: Prediction of preeclampsia risk in first time pregnant women: Metabolite biomarkers for a clinical test
Source: PLoS One. 2020 Dec 28;15(12):e0244369. doi: 10.1371/journal.pone.0244369 (PMC7769282; doi:10.1371/journal.pone.0244369)
Supplement: S6 Table — (DOCX) [file pone.0244369.s011.docx]

**S6 Table**. **Quantification metric selection and assay selection for biomarker analyses.**

| **Assay-able Metabolites** | **Assigned ISTD  *(dedicated SIL-IS)*** | **Batch Normalisation applied** | **Missing values** | **imprecision (%CV)** | | **Assay Selection counter** |
| --- | --- | --- | --- | --- | --- | --- |
|  |  |  | **MV > 20%** | **Study-QC pool**^a^ | **Replicates** |  |
|  |  |  |  |  |  |  |
| Isobutyrylglycine | N-Isobutyrylglycine [^13^C_2_, ^15^N] | NO | YES | N/A | N/A | N/A |
| Taurine | Taurine [^13^C_2_] | NO | NO | 9% | 8% | 1 |
| Urea | Urea [^13^C, ^18^O] | NO | NO | 7% | 7% | 2 |
| L-Palmitoylcarnitine | Palmitoyl carnitine-[^2^H_3_] | YES | NO | 13% | 12% | 3 |
| Stearoylcarnitine | Stearoyl-L-carnitine [^2^H_3_] | NO | NO | 14% | 10% | 4 |
| Decanoylcarnitine | Decanoylcarnitine-[^2^H_3_] | NO | NO | 13% | 15% | 5 |
| L-Acetylcarnitine | L-Acetylcarnitine [^2^H_3_] | NO | YES | 6% | 6% | 6 |
| Dodecanoyl-l-carnitine | Dodecanoyl-l-carnitine-[^2^H_3_] | NO | NO | 10% | 9% | 7 |
| 2-Methylglutaric acid + L-Glutamine | L-Glutamine [^13^C_5_] | YES | NO | 9% | 20% | N/A |
| Adipic acid | L-Glutamine [^13^C_5_] | YES | NO | 46% | 39% | N/A |
| 8,11,14 Eicosatrienoic acid | Arachidonic acid-[^2^H_8_] | NO | NO | 12% | 11% | 8 |
| 20-Carboxy-leukotriene B4* | Oleic acid-[^13^C_5_] | YES | NO | 18% | 17% | 9 |
| Eicosapentaenoic acid | Hexadecanoic acid-[^2^H_4_] | NO | NO | 19% | 26% | 10 |
| Oleic acid | Oleic acid-[^13^C_5_] | NO | NO | 10% | 9% | 11 |
| Linoleic acid | Linoleic acid-[^13^C_18_] | NO | NO | 10% | 8% | 12 |
| Docosahexaenoic acid | Docosahexaenoic acid-[^2^H_5_] | NO | NO | 12% | 11% | 13 |
| Ricinoleic acid | Linoleic acid-[^13^C_18_] | NO | YES | N/A | N/A | N/A |
| 13‑Oxooctadecanoic acid | Linoleic acid-[^13^C_18_] | NO | YES | N/A | N/A | N/A |
| Hexadecanoic acid | Hexadecanoic acid-[^2^H_4_] | NO | NO | 15% | 14% | 14 |
| Arachidonic acid | Arachidonic acid-[^2^H_8_] | NO | NO | 14% | 14% | 15 |
| Stearic acid | Linoleic acid-[^13^C_18_] | NO | NO | 20% | 20% | 16 |
| 2-Hydroxybutanoic acid | 2-Hydroxybutanoic acid ‑[^2^H_3_] | NO | NO | 8% | 8% | 17 |
| 3-Hydroxybutanoic acid | 3-Hydroxybutanoic acid [^2^H_4_] | NO | NO | 9% | 12% | 18 |
| 3‑Hydroxytetradecanoic acid | Linoleic acid-[^13^C_18_] | NO | YES | N/A | N/A | N/A |
| 2-Hydroxytetradecanoic acid | Oleic acid-[^13^C_5_] | NO | YES | N/A | N/A | N/A |
| Dilinoleoyl-glycerol^+^ | 1,3-Dilinoleoyl-rac-glycerol-[^2^H_5_] | NO | NO | 13% | 10% | 19 |
| 1-oleoyl-2-hydroxy-sn-glycero-3-phospho-L-serine |  | N/A | YES | N/A | N/A | N/A |
| (1,2-Dioctanoyl-sn-glycero-3-phosphocoline) 1-heptadecanoyl-glycero-3-phosphocholine | Sphingosine-1-phosphate-[^13^C_2_,^2^H_2_] | YES | NO | 16% | 13% | 20 |
| Sphingosine-1-phosphate | Sphingosine-1-phosphate-[^13^C_2_,^2^H_2_] | YES | NO | 16% | 11% | 21 |
| Sphinganine-1-phosphate | Sphingosine-1-phosphate-[^13^C_2_,^2^H_2_] | YES | NO | 16% | 16% | 22 |
| Sphinganine-1-phosphate (C17 base) | Sphingosine-1-phosphate-[^13^C_2_,^2^H_2_] | NO | YES | N/A | N/A | N/A |
| Bilirubin | Bilirubin [^2^H_4_] | YES | NO | 18% | 13% | 23 |
| Biliverdin | Biliverdin [^2^H_4_] | YES | NO | 20% | 21% | 24 |
| 25-Hydroxyvitamin D_3_ | 25-Hydroxyvitamin D_3_‑[^2^H_3_] | NO | NO | 26% | 21% | 25 |
| Etiocholanolone glucuronide | Oleic acid-[^13^C_5_] | NO | NO | 18% | 14% | 26 |
| L-Alanine | L-Alanine-[^13^C_3_] | NO | NO | 8% | 8% | 27 |
| L-Leucine | Leucine [^13^C_6_] | NO | NO | 8% | 7% | 28 |
| Choline | Choline-[^2^H_9_] | NO | NO | 8% | 7% | 29 |
| Glycyl-glycine | Glycyl-glycine [^13^C_4_, ^15^N_2_] | NO | NO | 31% | 30% | N/A |
| L-Isoleucine | Isoleucine-[^13^C_6_] | NO | NO | 9% | 6% | 30 |
| L-Methionine | L-Methionine-[^13^C_5_] | NO | NO | 7% | 7% | 31 |
| L-Lysine | L-Acetylcarnitine [^2^H_3_] | YES | NO | 8% | 6% | 32 |
| L-Glutamine | L-Glutamine [^13^C_5_] | NO | NO | 8% | 6% | 33 |
| L-Arginine | L-Arginine [^13^C_6_] | NO | NO | 8% | 7% | 34 |
| Citrulline | L-Citrulline [^2^H_7_] | NO | NO | 9% | 8% | 35 |
| Homo-L-arginine | Homo-L-arginine [^13^C_7_, ^15^N_4_] | NO | NO | 6% | 6% | 36 |
| NG-Monomethyl-L-arginine | Homo-L-arginine [^13^C_7_, ^15^N_4_] | NO | NO | 12% | 10% | 37 |
| Asymmetric dimethylarginine | Asymmetric dimethylarginine [^2^H_6_] | NO | NO | 8% | 8% | 38 |
| Symmetric dimethylarginine | Symmetric Dimethylarginine- [^2^H_6_] | NO | NO | 11% | 8% | 39 |
| 1-Palmitoyl-2-hydroxy-sn-glycero-3-phosphocholine | Linoleic acid-[^13^C_18_] | YES | NO | 10% | 9% | 40 |
| 6-Hydroxysphingosine** | Sphingosine-1-phosphate-[^13^C_2_,^2^H_2_] | NO | NO | 72% | 55% | N/A |
| Cotinine | (±)-Cotinine [^2^H_3_] | NO | Smoking | 10% | 20% | 41 |
| Myristic acid | Docosahexaenoic acid-[^2^H_5_] | YES | NO | 13% | 14% | 42 |
| L-(+)-Ergothioneine | L-(+)-Ergothioneine [^2^H_9_] | NO | NO | 19% | 12% | 43 |

N/A: Not applicable. ^a^ Coefficient of variability based on all pooled QC samples across all batches. Estimate of total technical variability accounting for sample preparation-, inter- and intra-batch variability. ^b^ Coefficient of variability based on duplicate plasma sample preparations (73 x 2); duplicates were randomized together with the other plasma samples. Estimate of total technical variability accounting for variability in sample matrix, in sample preparation, as well as inter- and intra-batch variability.
