# Supplementary material for: Prediction of preeclampsia risk in first time pregnant women: Metabolite biomarkers for a clinical test
Source: PLoS One. 2020 Dec 28;15(12):e0244369. doi: 10.1371/journal.pone.0244369 (PMC7769282; doi:10.1371/journal.pone.0244369)
Supplement: S7 Table — (DOCX) [file pone.0244369.s012.docx]

**S7 Table. Comparator predictors for the pre-set clinical use scenarios**

| **Primary Objective:**  **Prediction of increased risk of preterm preeclampsia at PPV >=0.05** | | | | | | |
| --- | --- | --- | --- | --- | --- | --- |
| **Model** | Method | NPV (95% CI) | **Specificity (95% CI)** | Sensitivity (95%CI) |  |  |
| **PlGF** | N/A | 0.059 (0.033-0.096) | **0.48** (0.26-0.70) | 0.89 (0.86-0.93) |  |  |
| **MAP** | N/A | ° | ° | ° |  |  |
| **BMI** | N/A | ° | ° | ° |  |  |
| **PlGF + MAP** | PLS-DA | 0.051 (0.020-0.093) | **0.30** (0.13-0.52) | 0.92 (0.89-0.95) |  |  |
| **PlGF + BMI** | PLS-DA | 0.121 (0.000-1.000) | **0.09** (0.00-0.22) | 0.99 (0.98-1.00) |  |  |
| **MAP +BMI** | PLS-DA | ° | ° | ° |  |  |
| **PlGF + MAP + BMI** | PLS-DA | ° | ° | ° |  |  |
| **PlGF \|\| MAP** | RP | 0.064 (0.038-0.100) | **0.52*** (0.30-0.74) | 0.89 (0.86-0.93) |  |  |
| **PlGF \|\| BMI** | RP | 0.058 (0.034-0.088) | **0.52***(0.30-0.70**)** | 0.88 (0.84-0.92) |  |  |
| **PlGF \|\| MAP + BMI** | RP & PLS-DA | 0.053 (0.034-0.079) | **0.57*** (0.39-0.78) | 0.86 (0.82-0.89) |  |  |
| **PlGF \|\| MAP \|\| BMI** | RP | 0.054 (0.036-0.078) | **0.61*** (0.43-0.78) | 0.85 (0.81-0.89) |  |  |
| **MAP \|\| BMI** | RP | 0.054 (0.012-0.116) | **0.22** (0.04-0.39) | 0.95 (0.92-0.97) |  |  |
| **Secondary Objective:**  **Prediction of decreased risk of any preeclampsia at NPV>=0.99** | | | | | | |
| **Model** | Method | NPV (95% CI) | **Specificity (95% CI)** | Sensitivity (95%CI) | |  |
| **PlGF** | N/A | 1.00 (1.00-1.00) | **0.02** (0.01-0.03) | 1.00 (1.00-1.00) | |  |
| **MAP** | N/A | 1.00 (0.99-1.00) | **0.13*** (0.09-0.17) | 0.99 (0.97-1.00) | |  |
| **BMI** | N/A | 1.00 (1.00-1.00) | **0.02** (0.01-0.03) | 1.00 (1.00-1.00) | |  |
| **PlGF + MAP** | PLS-DA | 1.00 (0.99-1.00) | **0.14*** (0.10-0.18) | 0.99 (0.97-1.00) | |  |
| **PlGF + BMI** | PLS-DA | 1.00 (1.00-1.00) | **0.02** (0.01-0.03) | 1.00 (1.00-1.00) | |  |
| **PlGF + MAP + BMI** | PLS-DA | 0.991 (0.975-1.000) | **0.12*** (0.09-0.16) | 0.98 (0.95-1.00) | |  |
| **PlGF \|\| MAP** | RP | 0.99 (0.98-1.00) | **0.16*** (0.13-0.21) | 0.97 (0.94-1.00) | |  |
| **PlGF \|\| BMI** | RP | 1.00 (1.00-1.00) | **0.02** (0.01-0.03) | 1.00 (1.00-1.00) | |  |
| **PlGF \|\| MAP + BMI** | RP & PLS-DA | 0.990 (0.981-0.998) | **0.28*** (0.23-0.32) | 0.95 (0.90-0.99) | |  |
| **PlGF \|\| MAP \|\| BMI** | RP | 0.990 (0.982-0.997) | **0.32*** (0.27-0.37) | 0.94 (0.89-0.98) | |  |
| **MAP \|\| BMI** | RP | 0.993 (0.981-1.000) | **0.15*** (0.11-0.19) | 0.98 (0.95-1.00) | |  |

* significantly higher compared to PlGF (T test, p<0.05, Bonferroni multiple testing correction); °: sensitivity null at PPV= 0.05. CI confidence interval; DLG= Dilinoleoyl-glycerol; 1-HGP= 1-heptadecanoyl-2-hydroxy-sn-glycero-3 phosphocholine; RP= recursive partitioning; PLS-DA= partial least squares discriminant analysis. N/A= not applicable
