# Supplementary material for: Prediction of preeclampsia risk in first time pregnant women: Metabolite biomarkers for a clinical test
Source: PLoS One. 2020 Dec 28;15(12):e0244369. doi: 10.1371/journal.pone.0244369 (PMC7769282; doi:10.1371/journal.pone.0244369)
Supplement: S1 Fig — (DOCX) [file pone.0244369.s013.docx]

**S1 Fig: Partitioning rule-in model (PlGF || DLG + 1-HGP).**


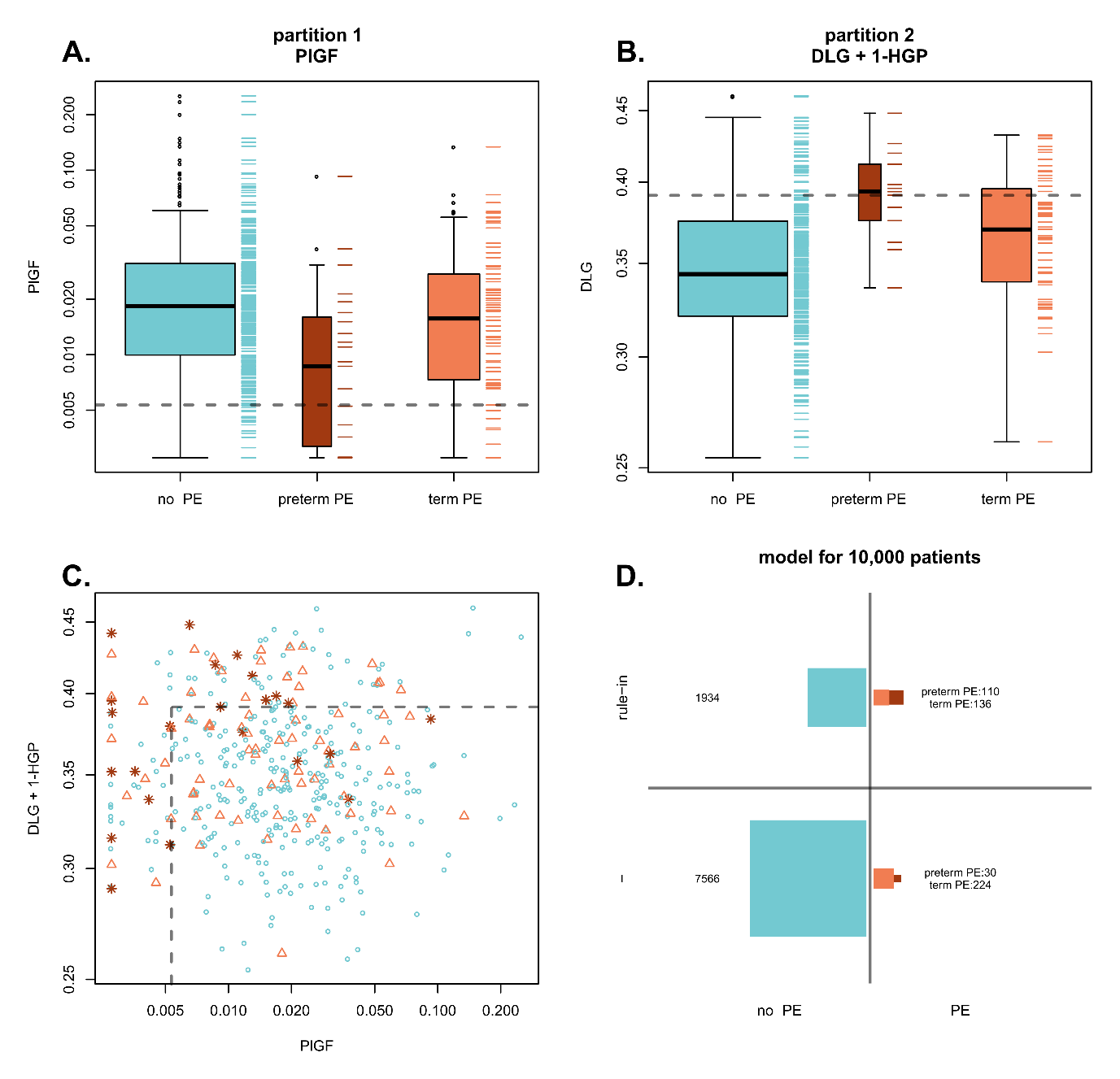


The predictive performance of this model is sensitivity 0.78 (0.61−0.96), PPV 0.052 (0.039−0.069) for preterm preeclampsia. The sensitivity of this model is significantly superior to that of model PlGF || DLG (p<0.001, T test). (A) Partition 1: PlGF concentration (µg/mL) versus clinical outcome. PlGF cut-off value = 0.00526 (dotted line). (B) Partition 2: DLG + 1-HGP risk score versus clinical outcome for patients ruled-out in partition 1. DLG + 1-HGP cut-off value = 0.39 (dotted line). (C) Partitions 1 and 2: DLG + 1-HGL risk score versus PlGF concentration (red stars: preterm preeclampsia, orange triangles: term preeclamspia, blue circles: no preeclampsia). Dotted line: DLG + 1-HGP and PlGF cut-off values. (D) Estimated predictive performance of the model for 10,000 patients. The DLG + 1-HGP risk score is a PLS-DA model (one component) trained on patients with PlGF concentration higher or equal to that corresponding to the observed maximum accuracy for the prediction of preeclampsia.
