# Supplementary material for: Prediction of preeclampsia risk in first time pregnant women: Metabolite biomarkers for a clinical test
Source: PLoS One. 2020 Dec 28;15(12):e0244369. doi: 10.1371/journal.pone.0244369 (PMC7769282; doi:10.1371/journal.pone.0244369)
Supplement: S2 Fig — (DOCX) [file pone.0244369.s014.docx]

**S2 Fig: Biomarker concentrations in function of gestational age at blood sampling.**


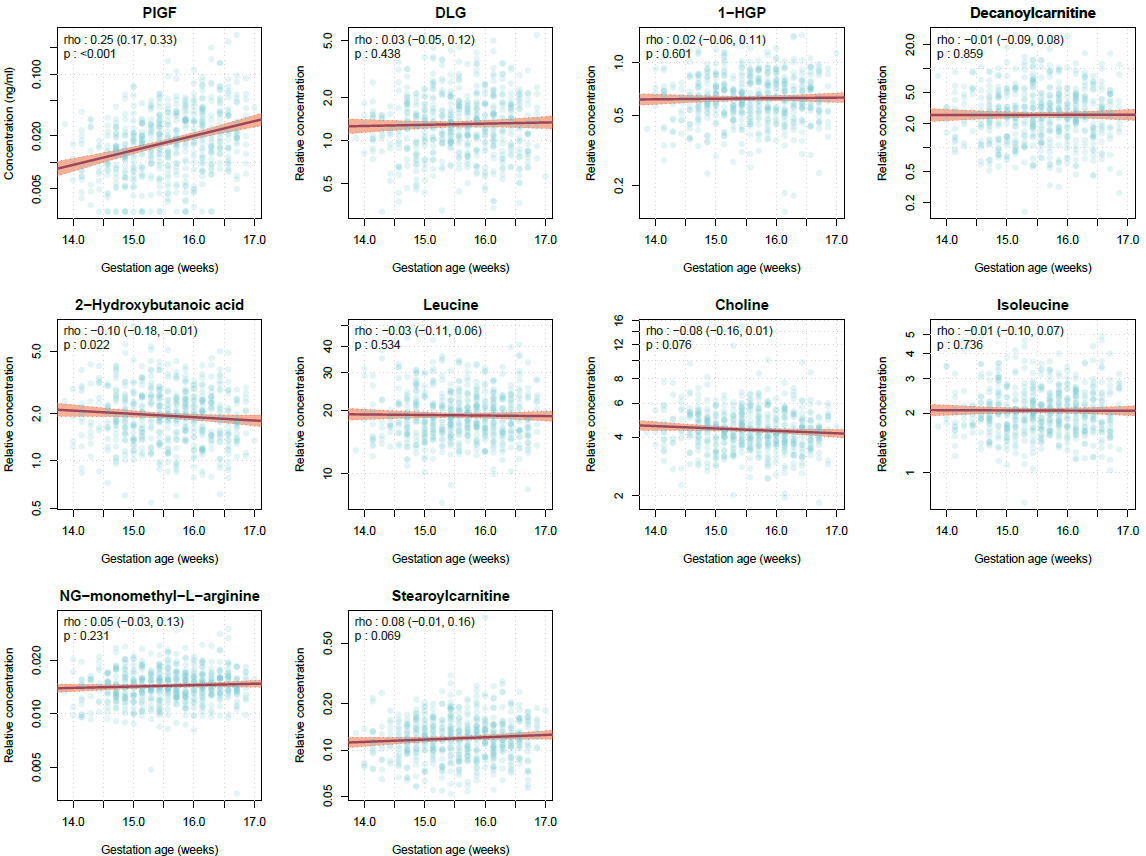


Biomarker levels are log-transformed. Correlation estimated using Pearson's correlation coefficient rho (95% confidence interval) and p-value.
